# Establishment of a risk model by integrating hypoxia genes in predicting prognosis of esophageal squamous cell carcinoma

**DOI:** 10.1002/cam4.5002

**Published:** 2022-07-04

**Authors:** Wanyi Xiao, Peng Tang, Zhilin Sui, Youming Han, Gang Zhao, Xianxian Wu, Yueyang Yang, Ningning Zhu, Lei Gong, Zhentao Yu, Hongdian Zhang

**Affiliations:** ^1^ Department of Esophageal Cancer Tianjin Medical University Cancer Institute and Hospital, Key Laboratory of Cancer Prevention and Therapy of Tianjin, Tianjin's Clinical Research Center for Cancer, National Clinical Research Center for Cancer Tianjin China; ^2^ Department of Thoracic Surgery National Cancer Center, National Clinical Research Center for Cancer, Cancer Hospital & Shenzhen Hospital, Chinese Academy of Medical Sciences and PeKing Union Medical College Shenzhen China; ^3^ Department of Respiratory Medicine Binhai Hospital of Tianjin Medical University General Hospital Tianjin China; ^4^ Department of Gastrointestinal Cancer Biology Tianjin Medical University Cancer Institute and Hospital, Key Laboratory of Cancer Prevention and Therapy of Tianjin, Tianjin's Clinical Research Center for Cancer, National Clinical Research Center for Cancer Tianjin China

**Keywords:** esophageal squamous cell carcinoma, hypoxia, hypoxia‐related genes, nomogram, prognosis

## Abstract

**Background:**

Esophageal squamous cell carcinoma (ESCC) has a dismal prognosis, and hypoxia plays a key role in metastasis and proliferation of ESCC. Thus, we aimed to develop a hypoxia‐based gene signature to assist in the treatment decisions and prognosis.

**Methods:**

We performed consensus clustering analysis on samples from GSE53625 dataset from the Gene Expression Omnibus (GEO) database and used weighted gene co‐expression network analysis to filter out candidate modules, which were then intersected with differentially expressed genes from clustered subgroups to obtain hypoxia‐related genes (HRGs). After that, the aforementioned genes were used to construct risk score models and validated in The Cancer Genome Atlas (TCGA) database and Cox regression analysis were used to construct a nomogram. Immunohistochemical was used to detect protein expression levels of relevant genes. Moreover, the relationship between risk scores and tumor microenvironment was explored.

**Results:**

A hypoxia risk model containing six genes (PNPLA1, CARD18, IL‐18, SLC37A2, ADAMTS18, and FAM83C) was constructed by screening key HRGs. Poorer prognosis in the high‐risk group than in the low‐risk group. And Cox regression analysis showed that risk score was an independent prognostic factor. The nomogram based on risk scores could well predict 1‐, 3‐, and 5‐year survival. P53, Wnt, and hypoxia signaling pathways may be some regulatory mechanisms of hypoxia associated with the tumor microenvironment. In addition, we confirmed the high expression of BGN and low expression of IL‐18 in ESCC tissues.

**Conclusions:**

Our study determined the prognostic value of a 6‐hypoxia gene signature and a prognostic model, providing potential prognostic predictors and therapeutic targets for ESCC.

## Graphical abstract

Our study systematically explored HRGs and determined the prognostic value of a 6‐hypoxia gene signature. A prognostic model was developed and validated based on this, providing potential prognostic predictors and therapeutic targets for ESCC

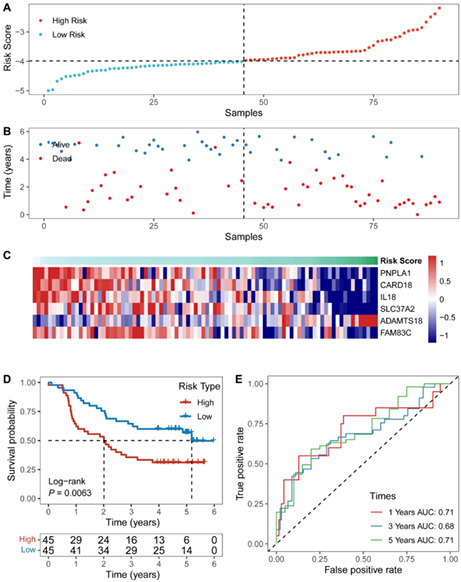

## 1 INTRODUCTION

Esophageal squamous cell carcinoma (ESCC) is the predominant type of esophageal cancer in China, accounting for more than 90% of pathological types.^1^ Due to the heterogeneous biological characteristics of ESCC,^2^ various mechanisms promote tumor progression leading to poor prognosis.

Rapid growth and massive angiogenesis increase oxygen consumption,^3^ resulting in hypoxia. Hypoxia is closely related to abnormal biological behaviors, leading to aberrant gene expression changes,^4^ anti‐apoptosis,^5^ suboptimal treatment outcomes,^6^ and ultimately to poor prognosis as tumor cells adapt to hypoxia.^7^ It has been found that ESCC tissues are invariably hypoxic. Hypoxia is involved in the migration and progression of ESCC and is associated with increased malignancy and poor prognosis.^8^ And hypoxia plays a crucial role in subsequent proteomic changes were reported.^9^ Therefore, focusing on hypoxia‐related markers may provide an efficient identification of specific patient groups.

With advances in bioinformatics and high‐throughput technologies,^10^ analyses that target the regulation networks are improving the current understanding of the molecular mechanisms. Since tumor hypoxia cannot be predicted based on clinical size, stage or differentiation, molecular biomarkers that can assess the hypoxic status of ESCC at an early stage and predict the prognosis are needed.

It has been reported that hypoxia and the tumor immune microenvironment also interact with each other.^11^ The adaptation of tumor cells to the hypoxic environment leads not only to aberrant gene expression but also to the formation of the tumor immune microenvironment. Exploring the effects of hypoxia on both and the potential remodeling effects will help reveal the complex interactions among them and provide new clues for the treatment of ESCC.

In this study, we aimed to identify and validate hypoxia‐related genes (HRGs) in ESCC and develop a nomogram based on the HRG signature to assist in the prognostic risk prediction of patients and to facilitate personalized treatment.

## 2 MATERIALS AND METHODS

### 2.1 Data acquisition and processing

We downloaded clinical information, including follow‐up data of 179 ESCC and 179 paired normal control samples from the Gene Expression Omnibus (GEO, GSE53625 dataset) based on GPL18109. Additionally, the RNA‐FPKM data and clinical data of 82 ESCC samples were retrieved for subsequent external validation analysis using The Cancer Genome Atlas (TCGA) data portal. Corresponding normal samples included 11 TCGA paracancerous samples and 1445 samples of 54 noncancer sites in GTEx. The median value was calculated when more than one expression file matched patients.

Tissue microarrays (TMAs) made by Shanghai Outdo Biotech Co., Ltd. were used for immunohistochemical staining (IHC). Samples of 232 ESCC tissues and 58 adjacent normal esophageal tissues with reliable survival information were obtained between 2009 and 2010 at Tianjin Medical University Cancer Institute and Hospital. Patients who had received neoadjuvant chemotherapy or radiotherapy and those with cancer types other than ESCC were excluded. Specimens were taken from the center of the tumor. Paired normal tissues were taken from surgically dissected tissues ∼5 cm away from the tumor. Among the patients, there were 192 males and 40 females with a median age of 67 years old. All patients were followed up until September 2016 with a median survival of 31 months. Enrolled patients authorized the collection and use of his or her material and signed written informed consent forms in advance. Ethical approval was obtained from the Research Ethics Committee of Tianjin Medical University Cancer Institute and Hospital, and the ethical number was bc2021340.

### 2.2 Extraction of HRGs

The list of 112 hypoxia genes was retrieved by enquiring “hypoxia” from The Cancer Single‐Cell State Atlas (CancerSEA)^12^ of all cancer types. The “HALLMARK‐HYPOXIA” gene set was derived from The Molecular Signatures Database (MSigDB).^13^ A total of 270 unique elements were subsequently obtained by intersecting the abovementioned hypoxia gene sets from the two sources and were identified as key genes involved in hypoxia activity.

### 2.3 Differential analysis of gene expression

Differentially expressed genes (DEGs) were determined using the “limma” package in R software, accounting for the nonindependence of samples from the same participant using limma's duplicateCorrelation. Multiple comparisons were adjusted using the Benjamini–Hochberg false discovery rate (FDR). An FDR <0.05 and a log2 |fold change| >2 were deemed as cutoff values for differentially expressed genes (DEGs).

### 2.4 Identification of hypoxia subgroups

To identify distinct subgroups of ESCC for optimal classification purposes, Euclidean‐based consensus clustering was performed on the GSE53625 dataset by the K‐means algorithm using the ConsensusClusterPlus R package. The specific parameters were clusterAlg = “km”, distance = “euclidean, corUse” = “everything”, innerLinkage = “ward.D2”. The clustering process was performed 500 times with each iteration containing 80% of the samples and the number of clusters set to 2–10. Each algorithm was run, and the consensus values and the stability of the clustering results were assessed by applying the given clustering method to random subsets of data.

After executing ConsensusClusterPlus, the graphical output results included heatmaps of the consensus matrices, which displayed the clustering results, consensus cumulative distribution function (CDF) plots, and Delta area plots. The optimal number of clusters is usually chosen as the value of K corresponding to the last inflection point of the CDF and the slightest slope of Delta.

### 2.5 Weighted gene coexpression network analysis (WGCNA)

The GSE53625 gene expression file for 179 ESCC samples was used to construct a scale‐free network using the R package “WGCNA”.^14^ Scale‐free R^2^ ranging from 0 to 1 was used to determine a scale‐free topology model. To minimize the effects of noise and spurious associations, the adjacency matrix was transformed into a topological overlap matrix (TOM), which was used to form modules by dynamic tree cut. We set the minimal module size to 20 and the cut height to 0.25.

### 2.6 Construction and evaluation of the prognosis prediction model

The stepAIC algorithm running in the R “MASS” package was used to construct an optimal prognostic model, based on the combination of expression profiles that intersected from selected modules and DEGs and prognostic information from 179 ESCC samples. The risk score of each sample was calculated according to the expression levels of the samples and then divided into two groups according to the median value after sorting. Half of the samples were randomly taken as the training group to construct the model. In contrast, the other half (internal validation set) and all of the GSE53625 datasets were used as testing datasets to assess the robustness of the model. The same coefficient was used for the external validation dataset ‐ TCGA. Kaplan–Meier curve analysis was further conducted to evaluate the relationship between the risk score and overall survival. The area under the curve (AUC) of the receiver operating characteristic (ROC) curves was calculated using the “time ROC R" package. The AUC value greater than 0.6 means the excellent predictive performance of this model.

### 2.7 Determining the classification features using Cox proportional risk regression models

In this study, significant prognostic variables obtained from the uni‐ and multivariate Cox regression model were then introduced into the final nomogram model. Calibration curves were plotted, and higher overlap with the 45‐degree standard curve indicated better predictive agreement. The “rms”, “foreign” as well as “survival” packages were used for nomogram construction and calibration curve plotting.

### 2.8 Protein–protein interaction (PPI) network and functional analysis

STRING website (http://string‐db.org/) was used to explore the protein interaction relationship of hypoxia‐related DEGs based on the PPI network. We used the “clusterProfiler” R package for Kyoto Encyclopedia of Genes and Genomes (KEGG) pathway enrichment analysis and for Gene Ontology (GO) analysis with respect to three domains: Cellular component (CC), biological process (BP), and molecular function (MF).

### 2.9 Implementation of gene set enrichment analysis (GSEA)

To further explore biological signaling pathways between differentially activated consensus subgroups and the high/low‐risk groups, GSEA was conducted using GSEA software employing GSE53625 data. *p* < 0.05 and a *Q* value less than 0.25 were considered to denote significant enrichment.

### 2.10 The ESTIMATE and CIBERSORT algorithms

The stromal, immune, and ESTIMATE score for each patient was calculated through the R “estimate” package. The fraction of 22 immune cell types was assessed through cell type identification (CIBERSORT; https://cibersort.stanford.edu/).

### 2.11 Immunohistochemical (IHC) analysis

TMAs were used for IHC to examine the protein expression level of selected HRGs. In brief, the tissue sections were deparaffinized (70°C, 2 h), rehydrated, and subjected to antigen repair with heated antigen retrieval solution (10 mmoL/L sodium citrate buffer, pH 6.0) (100°C, 10 min). The activity of endogenous peroxidase was blocked with 0.3% H_2_O_2_ and 5% goat serum. Then, the sections were incubated with primary antibodies (1:50, BGN, Proteintech; 1:100, IL‐18, ORIGENE) overnight at 4°C and then incubated with a biotinylated secondary antibody for 20 min at room temperature. Diaminobenzidine (Zhongshan Inc.) was used as a chromogen and produced a brown color, and then samples were counterstained with hematoxylin.

Two experienced pathologists who were blinded to the clinical data scored the staining results. Staining was assessed according to the intensity of staining (no staining, 0; weak, 1; moderate, 2; and strong, 3) and the percentage of positively stained tumor cells (0%, 0; 1%–30% positive, 1; 31%–70% positive, 2; 71%–100% positive, 3). A total score of 0–9 was obtained by multiplying the results of the staining intensity and staining percentage scores, and tissues with a total score of 0–3 were considered to have low expression, while tissues with a score of 4–9 to have high expression.

### 2.12 Statistical analysis

Bioinformatics analysis and statistical analysis were conducted using R (version 4.0.2). Comparisons between two groups were presented via the Wilcoxon rank‐sum test and chi–squared test, while multiple comparisons were assessed via the Kruskal–Wallis test. The cutoff point of each subgroup was identified by the survminer package in R. Kaplan–Meier curves are presented between different subgroups, followed by the log‐rank test. ROC curves for 1‐, 3‐ and 5‐year survival were delineated to evaluate the predictive efficacy of the risk score. The *p*‐values were corrected by Bonferroni's test. A two‐sided *p* < 0.05 was considered statistically significant.

## 3 RESULTS

### 3.1 Screening differentially expressed hypoxia genes

**FIGURE 1:**
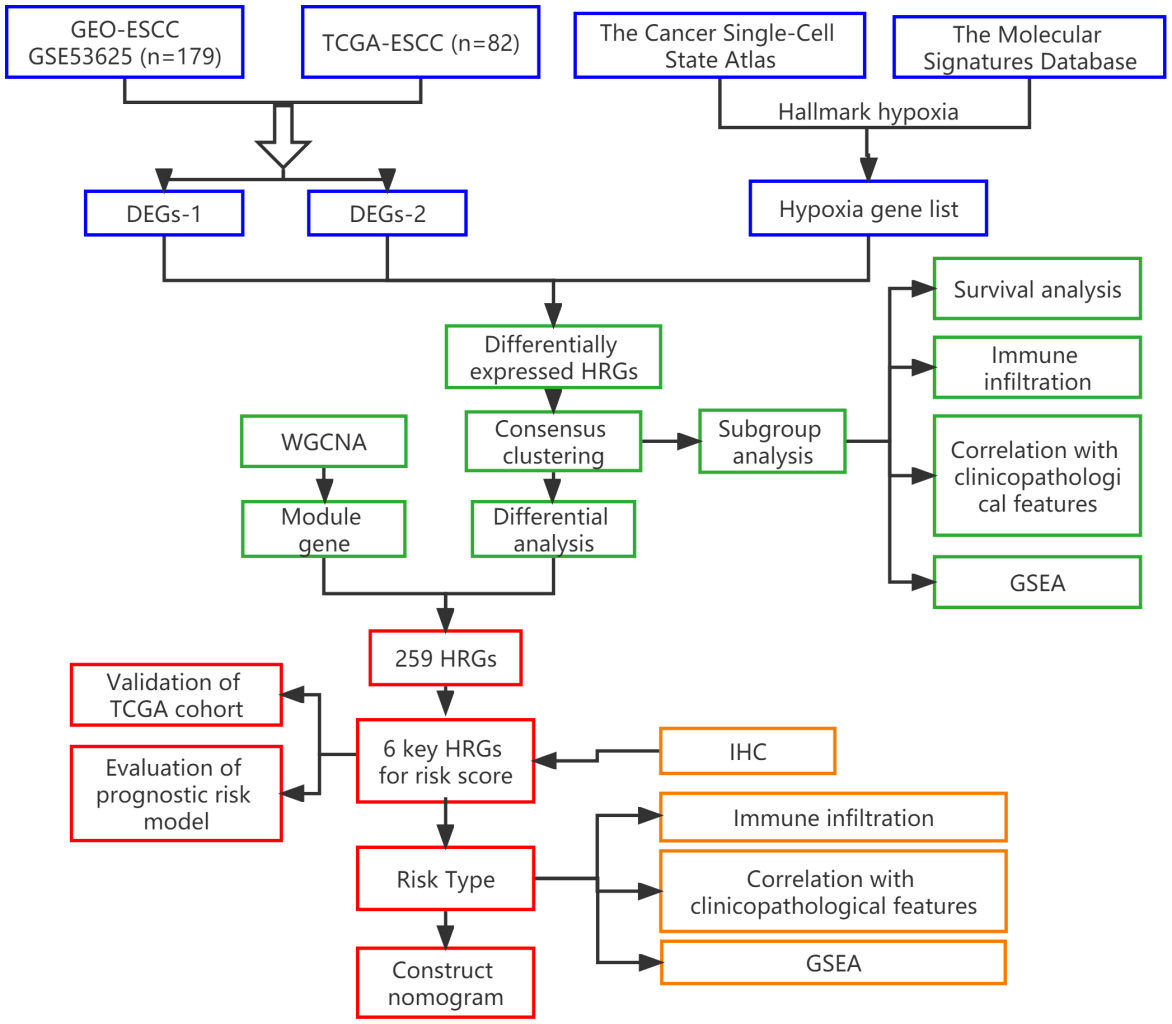
The flowchart for establishing the HRG signature of ESCC in the study. ESCC, esophageal squamous cell carcinoma; DEGs, differentially expressed genes; HRGs, hypoxia‐related genes; GEO, gene expression omnibus; TCGA, the cancer genome Atlas; IHC, immunohistochemical staining; GSEA, gene set enrichment analysis; WGCNA, weighted gene coexpression network analysis.

**FIGURE 2:**
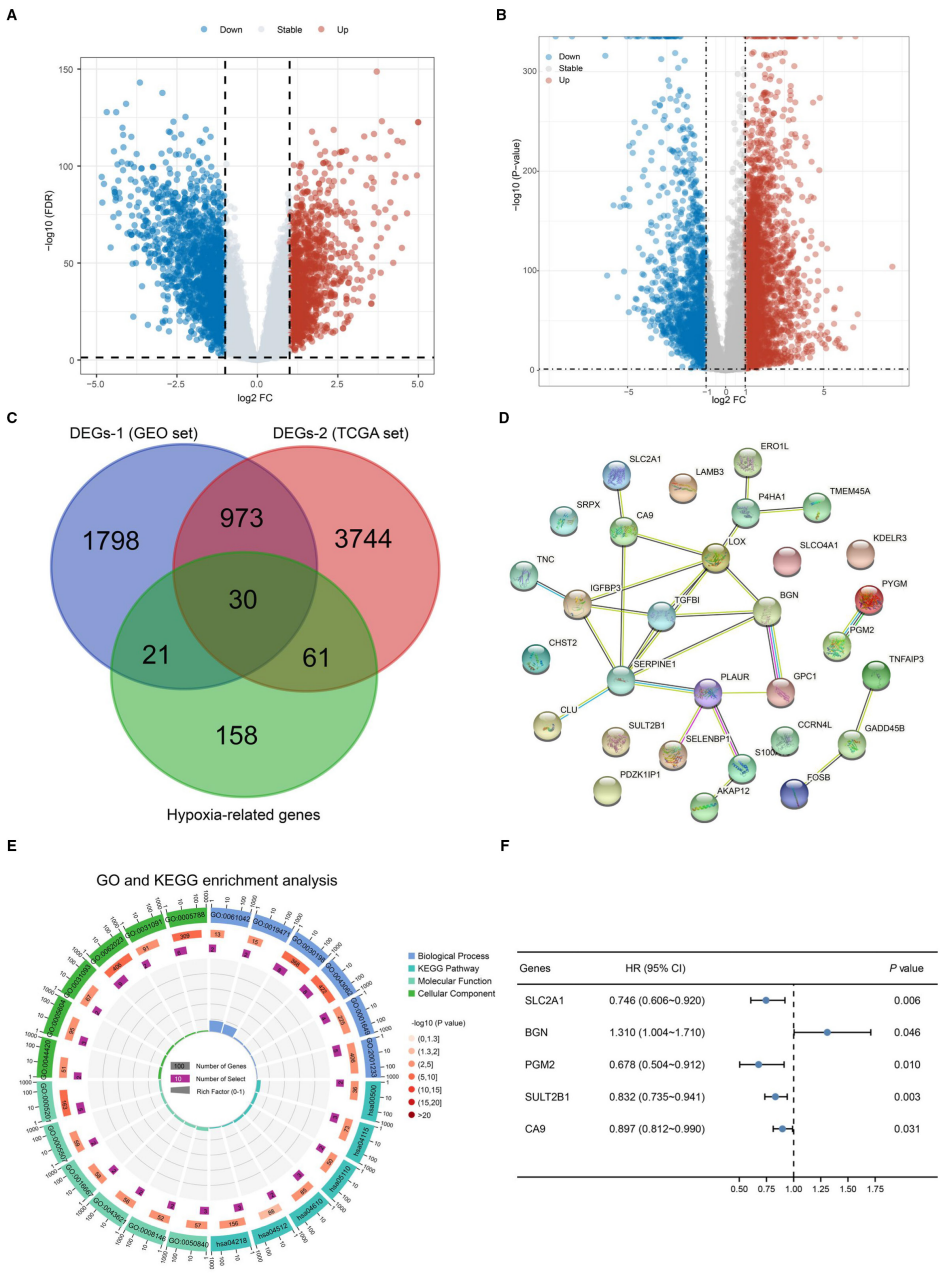
Identification of potential HRGs and related functional analysis. Differential expression analysis in ESCC (A–B). Volcano plots showing DEGs in (A) GES53625 and (B) TCGA‐ESCC. (C) Venn diagrams of the 30 overlapping differentially expressed HRGs. (D) PPI network of the differentially expressed HRGs constructed in STRING and visualized by Cytoscape. (E) The top 10 significant terms from the GO and KEGG analyses of differentially expressed HRGs. (F) Screening for potential HRGs of prognostic significance by univariate COX analysis.

All the procedures are displayed in the flowchart (Figure 1). After data preprocessing, 2822 DEGs in the GEO set and 4808 DEGs in TCGA were obtained. These are illustrated in the volcano plots in Figure 2A,B and heatmaps in Figure S1. We then intersected the DEGs with the list of 270 HRGs as shown in the Venn diagrams in Figure 2C, 30 hypoxia genes (15 upregulated and 15 downregulated) were identified (Table S1).

A subsequent PPI network was constructed by uploading the aforementioned 30 target genes (Figure 2D). In the functional analysis of DEGs, the top six enriched GO annotations and KEGG pathways in each category were visualized intuitively as Circos plots (Figure 2E). In the GO functional enrichment analysis, these 30 genes were mainly enriched in extracellular matrix and basement membrane in terms of CC, in extracellular matrix structural constituent and extracellular matrix binding in terms of MF and in the regulation of apoptotic signaling pathway in terms of BP. In the KEGG pathway enrichment analysis, these genes were mainly enriched in the P53 signaling pathway. The univariate survival analysis (*p* < 0.05, Figure 2F) showed that SLC2A1, PGM2, SULT2B1, and CA9 all had a lower risk of death except BGN.

### 3.2 Identifying distinct subgroups and intercluster prognosis analysis

**FIGURE 3:**
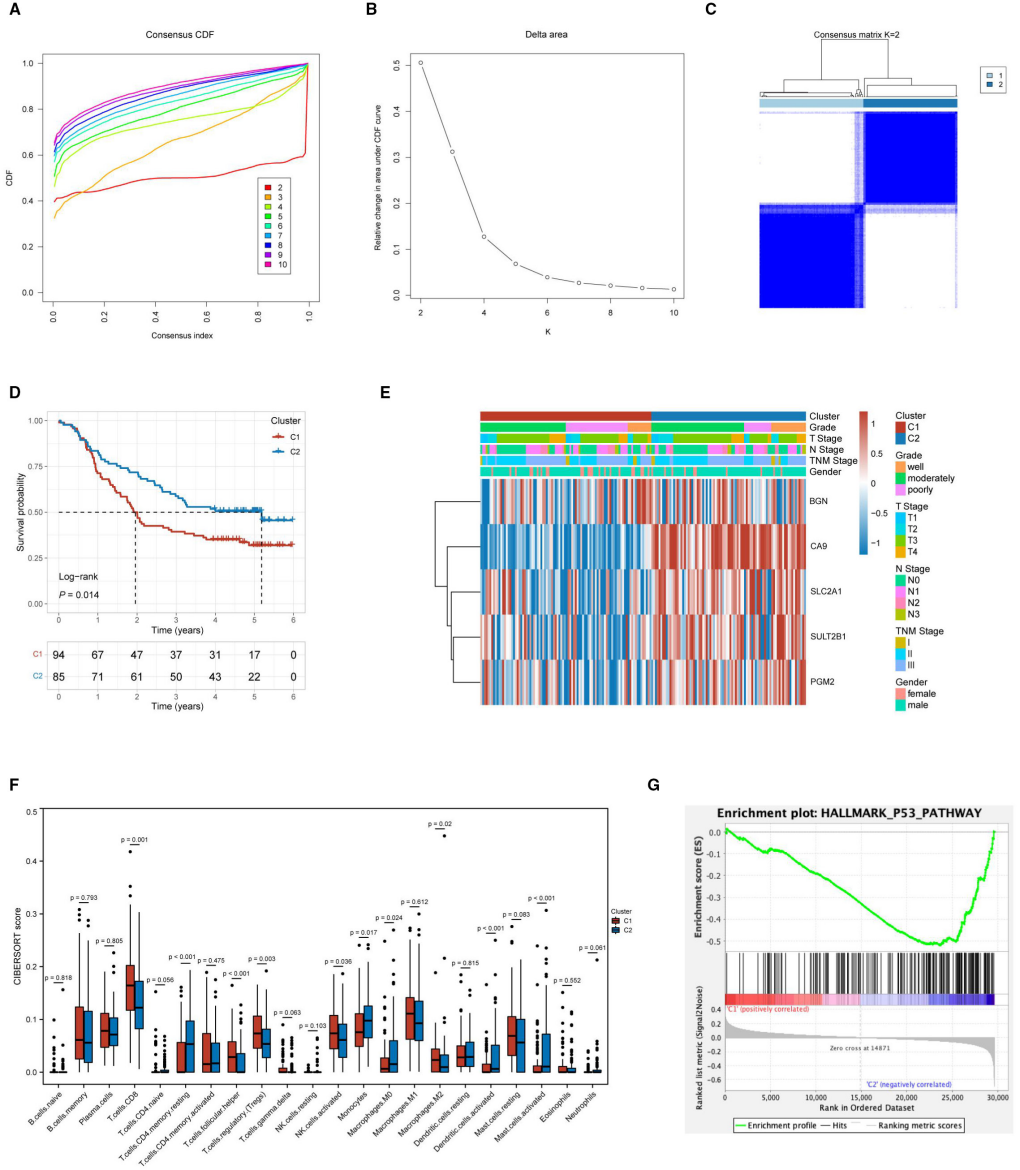
Analysis of subgroups with clinicopathological characteristics and immune infiltration. (A) Cumulative distribution map of clustering consistency. (B) Clustering Delta area map. (C) Display of the clustering results corresponding to K = 2. (D) The results of Kaplan–Meier survival analysis of different clusters. (E) A heatmap corresponding to the dendrogram annotated by grade, T stage, N stage, stage, sex, and five real hub genes. (F) The infiltration of 22 immune cell subtypes in different clusters. (G) GSEA was used to demonstrate the correlation between genes from different clusters and the KEGG enriched pathways.

A total of 179 tumor samples were consistently clustered based on the expression of screened hypoxia genes. The consensus matrix (Figure 3A,B) helps us to determine the most explicit division when divided into two clusters, named C1 and C2. Compared to other categorical numbers (Figure S2), the consensus matrix graph corresponding to K = 2 showed that the distribution of two blue blocks on the diagonal along the white background was well defined (Figure 3C).

The results of Kaplan–Meier survival analysis revealed significant differences between C1 and C2 (*p* = 0.014, Figure 3D). The OS of C2 was better than of C1. Sorting the samples by cluster produced the gene expression heatmap (Figure S3), which indicated the composition and quantity of clustering. Gene expression patterns differed among the subgroups, suggesting the credibility of the two structural clusters. A heatmap (Figure 3E) annotated by grade, T stage, N stage, stage, sex, and five real hub genes, demonstrated the heterogeneity between the two clusters.

### 3.3 Correlations between the obtained clusters and immune infiltration

We calculated the levels of 22 immune cell types in each sample and compared their differences in C1 and C2 (Figure 3F). The results showed that CD8^+^ T cells, resting memory CD4^+^ T cells, follicular helper T cells, regulatory T cells, activated NK cells, monocytes, M0 macrophages, activated dendritic cells, and activated mast cells had significantly different infiltration levels in different subgroups. Then, GSEA showed the activation of P53 signaling pathway molecules (Figure 3G).

### 3.4 Identification of modules associated with hypoxia

**FIGURE 4:**
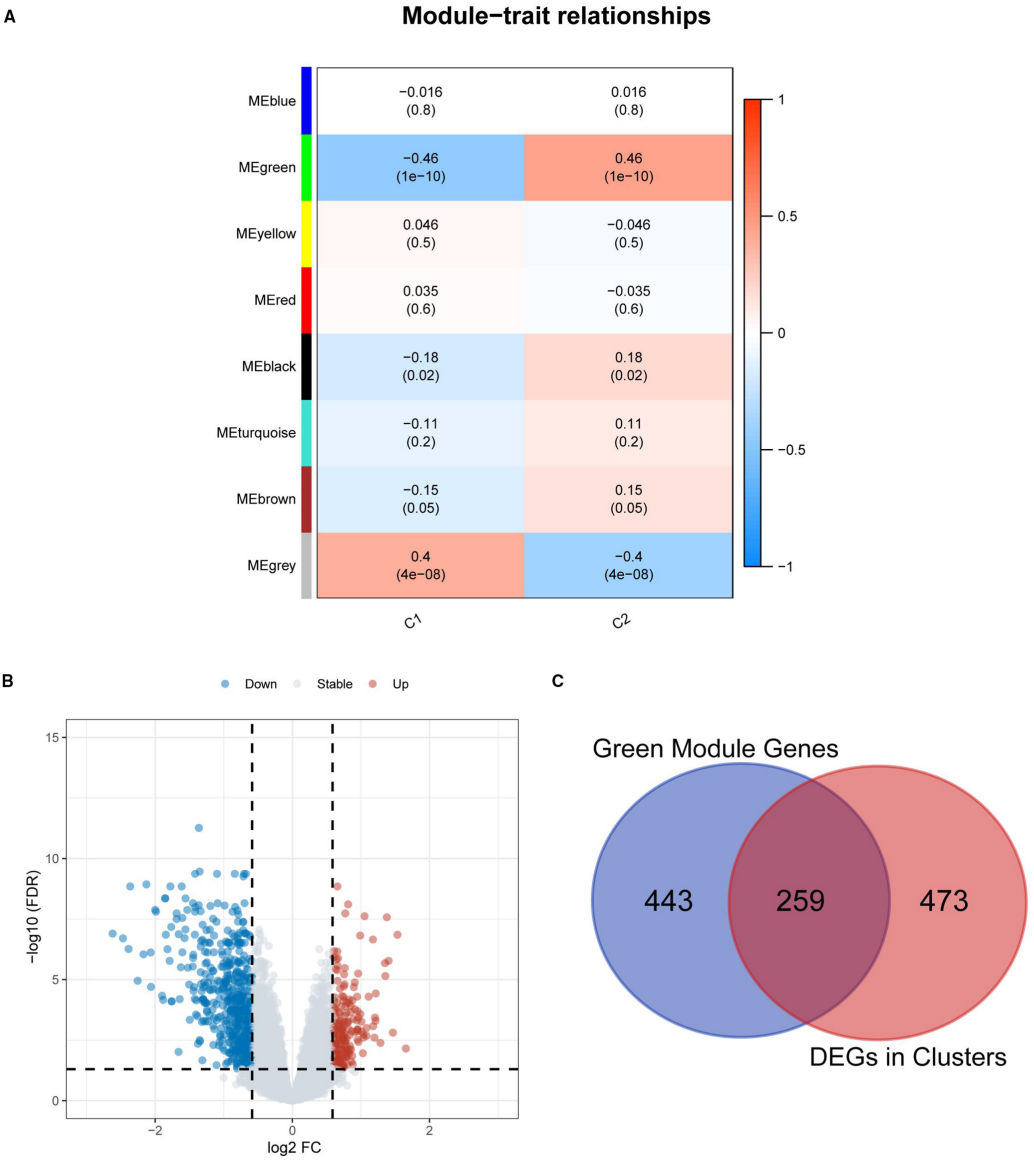
Screening of key HRGs. (A) The relationship of module features with consensus subgroups was assessed by eight gene modules obtained from WGCNA. (B) Volcano plots showing the DEGs in two clusters. (C) Venn diagrams of the 259 differentially expressed HRGs from intersection of green module genes and consensus clustering DEGs.

After sample clustering to detect outliers, the WGCNA was then restricted to 178 patients from GSE53625. Different power values 1~20 were analyzed, and the best power value of *β* = 5 (scale‐free R^2^ = 0.95) was chosen for soft thresholding for subsequent coexpression scale‐free network construction (Figure S4A–C). Eight gene modules were obtained and visualized for the following analysis (Figure 4A). The green module was significantly associated with consensus subgroups (C1, *r* = −0.46, *p* = 1e−10; C2, *r* = 0.46, *p* = 1e−10), suggesting that the module is suitable for identifying the hub genes associated with C1/C2.

### 3.5 Constructing and evaluating a hypoxia‐related prognosis signature

**FIGURE 5:**
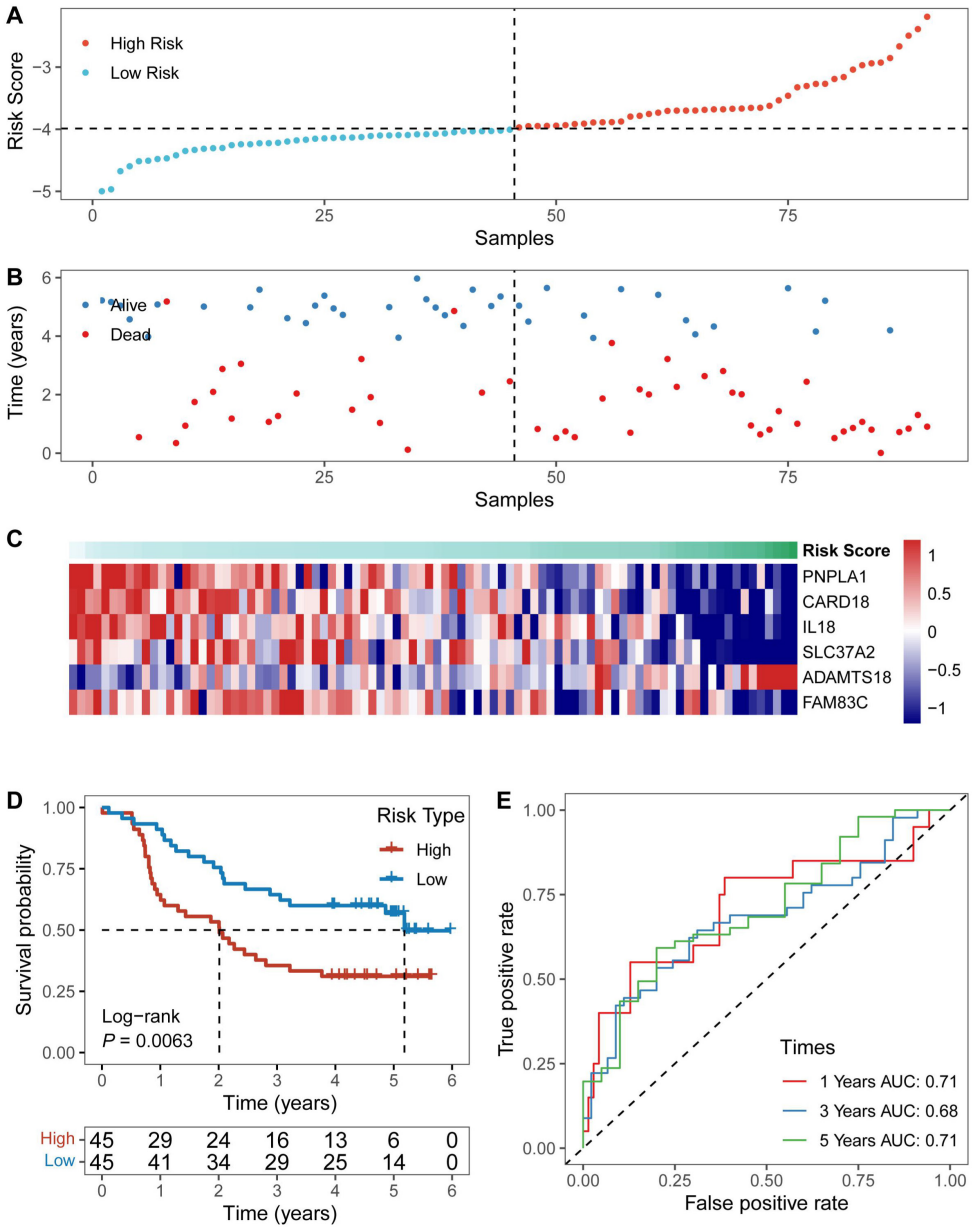
Constructing the prognostic hypoxia gene features in the training set. The samples were assigned a risk score and ordered to determine whether the expression level (A) and survival time (B) varied systematically with the risk score. (C) Expression levels of the 6 HRGs based on the risk score. (D) Survival curve distribution of the risk score. (E) ROC curves and AUCs of risk score classifications.

The results of differential expression analysis among the obtained clusters are shown in Figure 4B. The hypoxia gene signature was constructed based on 259 differentially expressed HRGs (Figure 4C), identifying the risk score using the 6 most relevant genes (Figure 5). The risk score was calculated as follows: Risk score = (0.066265 × PNPLA1 expression) + (−0.149270 × CARD18 expression) + (−0.183367 × IL‐18 expression) + (−0.037724079 × SLC37A2 expression) + (0.119388782 × ADAMTS18 expression) + (−0.031834954 × FAM83C expression). The samples were assigned a risk score and ordered to determine whether the expression level varied systematically with the risk score (Figure 5A‐C). A higher percentage of patient deaths was associated with high‐risk patients (*p* = 0.0063). Furthermore, the prognoses differed significantly between the two groups, as shown in Figure 5D. The results of the ROC curve are shown in Figure 5E. The AUCs for 1‐, 3‐, and 5‐year prognostic prediction were 0.71, 0.68, and 0.71, respectively, indicating the relatively excellent predictive efficacy of the model.

**FIGURE 6:**
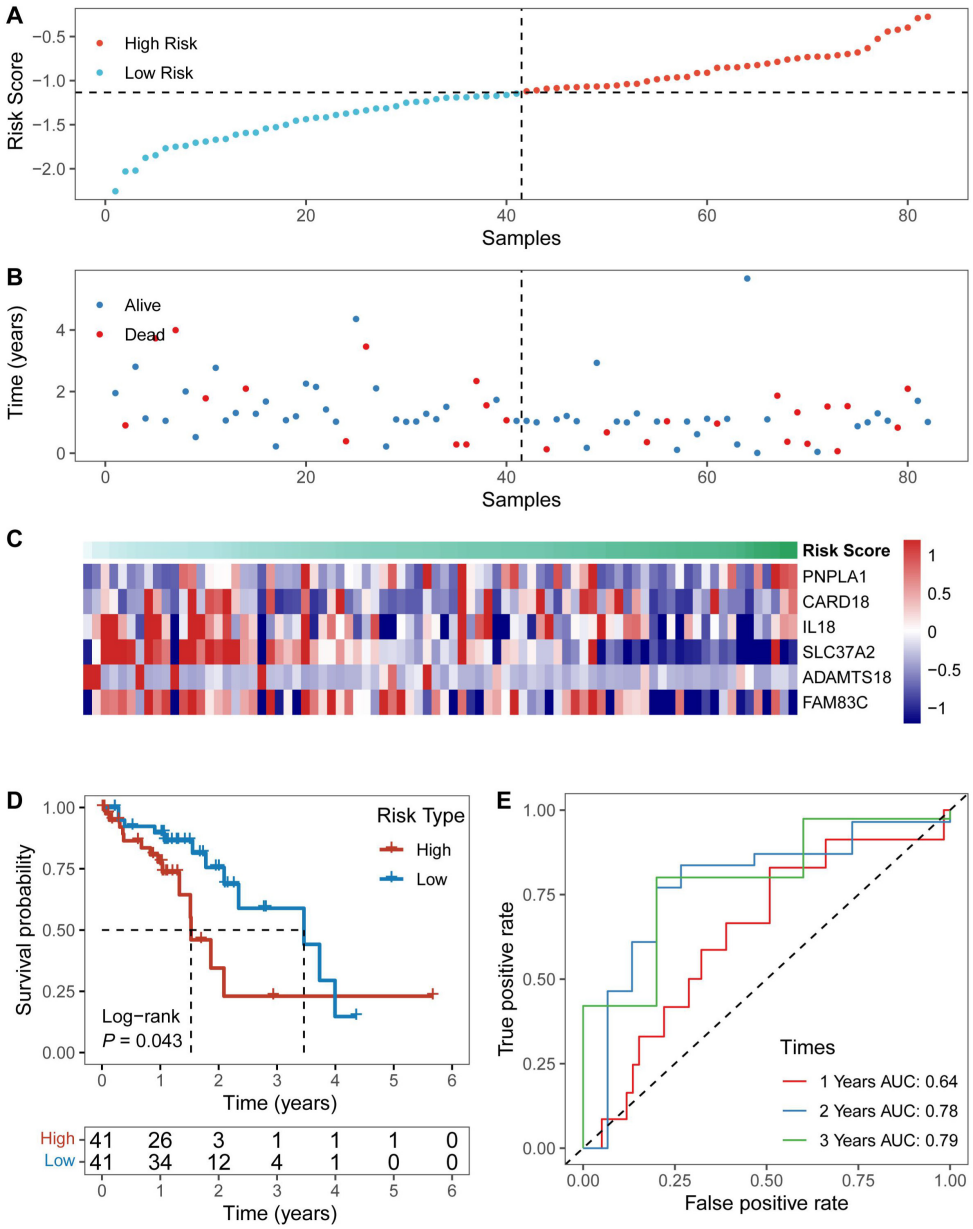
Validating the prognostic hypoxia gene features in the external TCGA dataset. The samples were assigned a risk score and ordered to determine whether the expression level (A) and survival time (B) varied systematically with the risk score. (C) Expression levels of the 6 HRGs based on risk scores. (D) Survival curve distribution of the risk score. (E) ROC curves and AUCs of risk score classifications.

Then, the established prognostic signature was further validated in the test group, including the internal validation set and all GSE53625 and TCGA datasets. Expression level profiles for the six selected genes were obtained from three testing group samples, and the risk scores were calculated using the abovementioned method for each patient. Sorting the samples by risk score produced the heatmap shown in Figure 6A–C, Figure S5A–C, and Figure S6A–C. The Kaplan–Meier survival curves showed that this risk model could effectively distinguish the survival (*p* = 0.0014 for the internal validation set, Figure S5D, *p* < 0.001 for all datasets, Figure S6D, and *p* = 0.043 for the TCGA dataset, Figure 6D). In the external validation group, the AUCs of the 1‐, 3‐, and 5‐year OS were 0.64, 0.78, and 0.79, respectively (Figure 6E). Consistent results are presented in Figure S5E and Figure S6E.

### 3.6 Establishment of predictive nomogram

**FIGURE 7:**
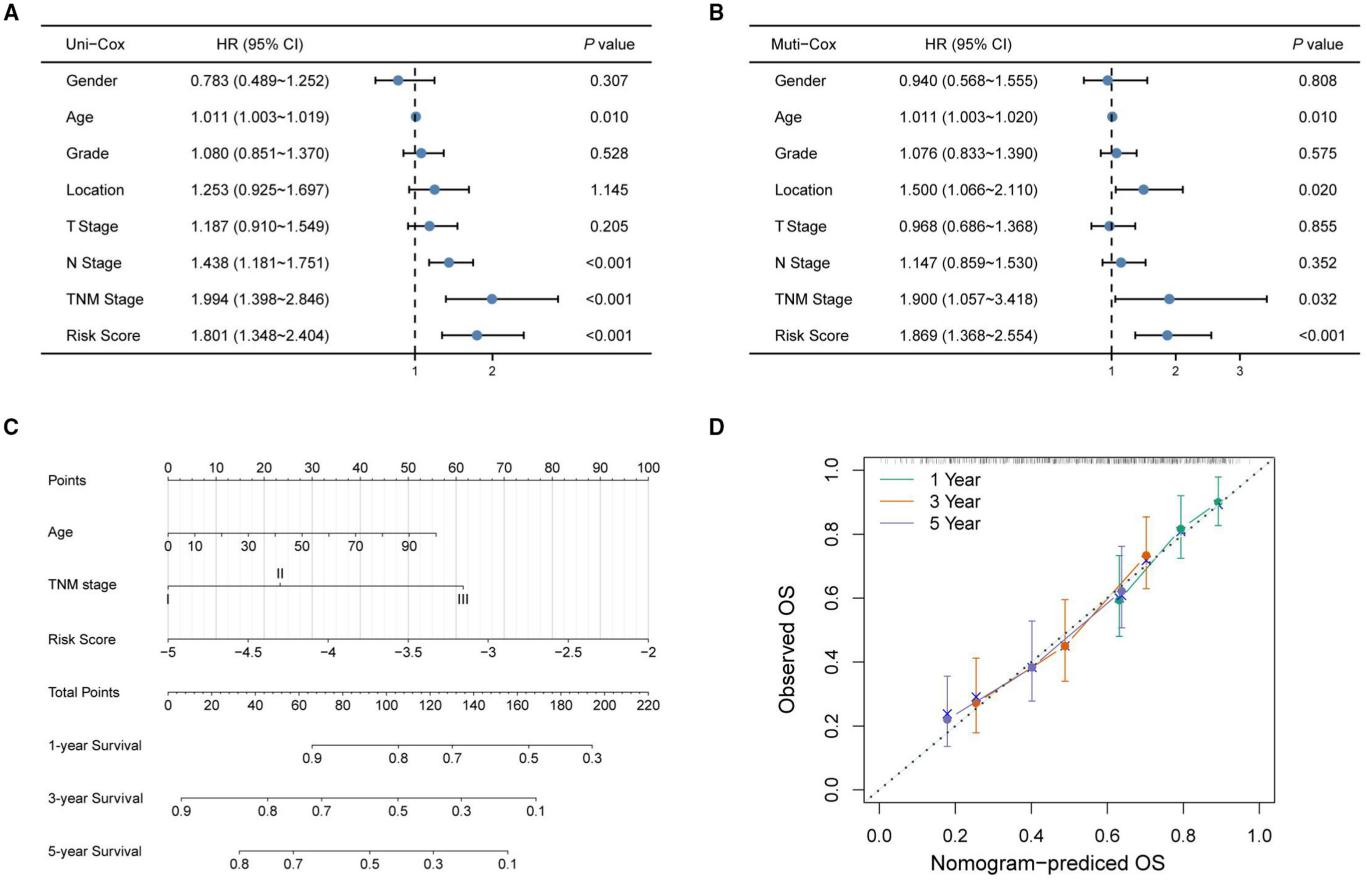
Evaluating the independent role of the prognostic signature and building a predictive nomogram. Univariate (A) and multivariate (B) Cox regression analyses were used to evaluate the predictive value of the model‐incorporated prognostic signature. (C) Integrating the risk score with age and stage to construct a nomogram model. (D) The calibration curve of the nomogram.

The univariate and multivariate Cox regression analyses were used to evaluate whether the predictive value of the model‐incorporated prognostic signature was affected by other clinical factors (Figure 7A,B). The results of multivariate Cox regression analyses indicated that age (*p* = 0.010, HR = 1.011), stage (*p* = 0.020，HR = 1.500), location (*p* = 0.032, HR = 1.900), and risk score (*p* < 0.001, HR = 1.869) were independent prognostic factors, and all are high risk factors. The risk score integrated with age and stage was chosen to construct a nomogram model, as presented in Figure 7C. The calibration plot of the nomogram (Figure 7D) showed better consistency between the predicted OS outcomes and actual observations, indicating the good predictive performance of the hypoxia‐related prognostic nomogram.

### 3.7 Correlation analysis of the risk score with clinicopathological features and immune infiltration

**FIGURE 8:**
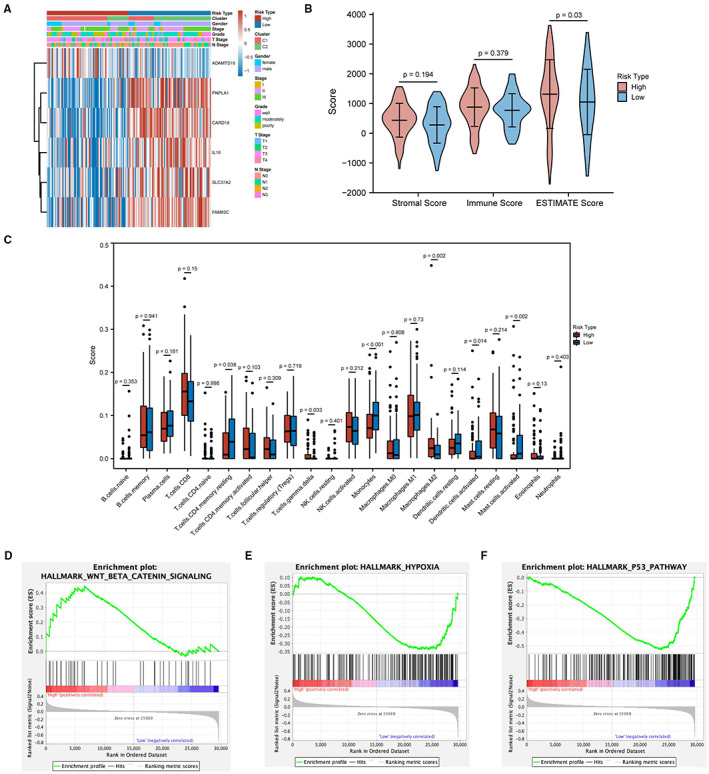
Analysis of the correlation between the risk score and immune infiltration. (A) Expression and clinical features of the 5 prognostic genes in the high‐ and low‐risk groups of the training set. (B) The results of the correlation analysis between the immune‐related score and the risk score. (C) The infiltration of 22 immune cell subtypes in the high‐ and low‐risk groups. (D–F) GSEA was used to demonstrate the correlation between HRG expression and the KEGG enriched pathways.

The high‐ and low‐risk groups were closely correlated with the clinical phenotypes, as shown by heat maps (Figure 8A). There was a significant difference in the degree of grade between patients in the high and low‐risk groups (*p* = 0.004), with a progressive increase in hypoxia score from well to poorly differentiation. It is suggested that a high hypoxia score is a detrimental factor for ESCC patients. The results (Figure 8B, Table S2) showed that the stromal and immune scores were comparable between subgroups. However, the estimated score was higher in the high‐risk group than in the low‐risk group, and the difference was statistically significant (*p* = 0.03). Differences in the infiltration of 22 immune cell subtypes between subgroups are shown in Figure 8C.

GSEA showed that the significantly enriched pathways were the P53 signaling pathway (Figure 8D), Wnt/β‐catenin pathway (Figure 8E), and hypoxia pathway (Figure 8F). The high‐risk group was positively correlated with the Hypoxia signaling pathway, while the low hypoxia risk group was negatively correlated with the Hypoxia signaling pathway, which also illustrates the accuracy of the risk grouping. In addition, the high‐risk group was positively correlated with the Wnt/β‐catenin signaling pathway, and the low‐risk group was negatively correlated with the P53 signaling pathway. The results reconfirmed that esophageal cancer cells in a hypoxic state could affect the tumor immune microenvironment through an underlying regulatory mechanism.

### 3.8 Validation of the expression of selected HRGs

**FIGURE 9:**
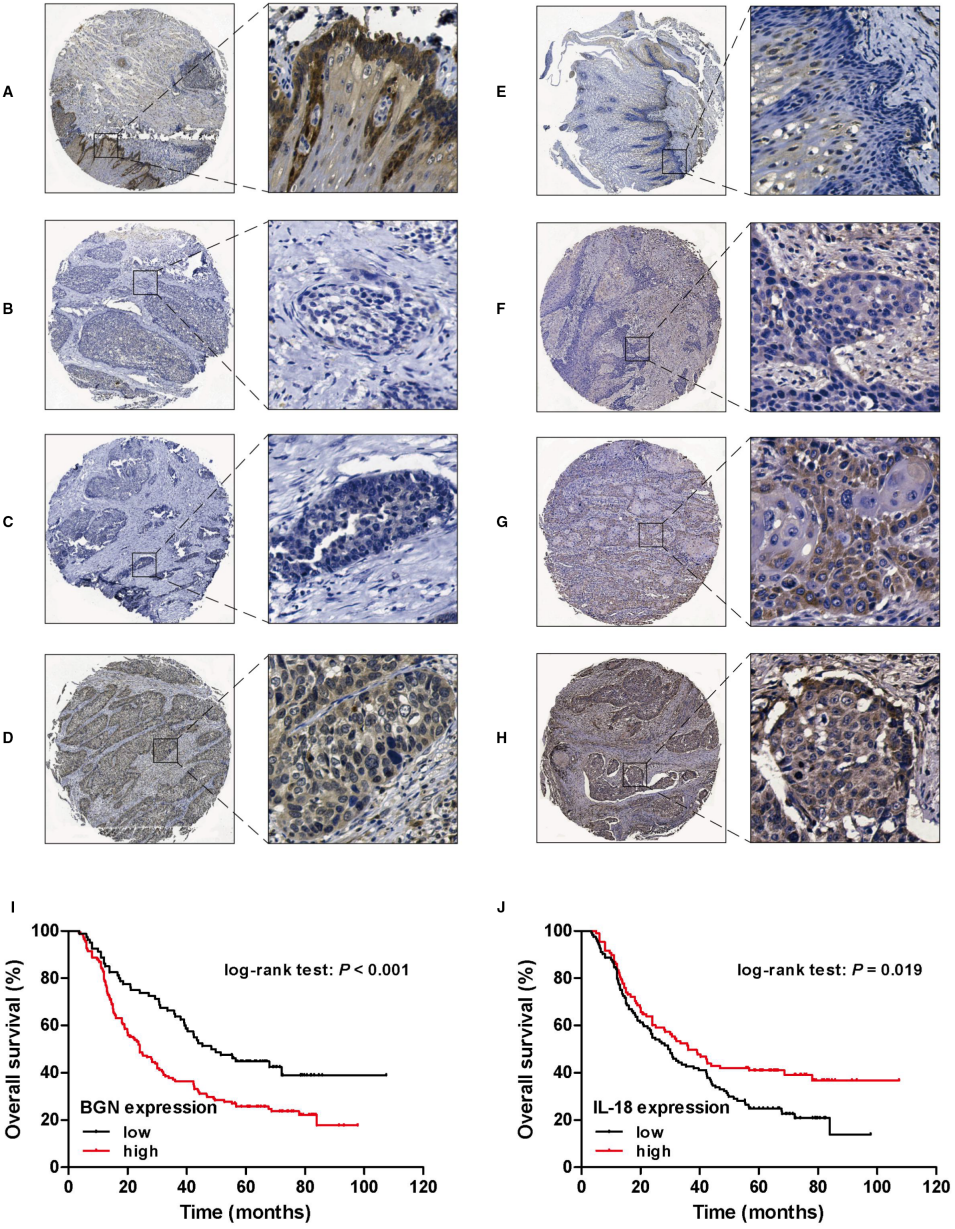
The expression of HRGs protein in ESCC and normal tissues was detected in TMA. Representative image of IHC staining for low expression of BGN in adjacent non‐tumor tissue (A) and low expression (B), high expression case 1 (C), and high expression case 2 (D) of BGN in human primary tumor tissue, stained 5x, inset 40x magnification. Representative image of IHC staining for high expression of IL‐18 in adjacent non‐tumor tissue (E) and low expression case 1 (F), low expression case 2 (G) and high expression (H) of IL‐18 in human primary tumor tissue, stained 5x, inset 40x magnification; (I) Kaplan–Meier curve of BGN expression in the TMA dataset. (J) Kaplan–Meier curve of IL‐18 expression in the TMA dataset.

To verify the accuracy of the abovementioned HRGs, we further detected the protein expression levels of BGN and IL‐18 according to previous publications and antibody availability. The clinical details of the 232 patients involved are presented in Table 1. BGN was highly expressed and mainly localized to the cytoplasm of cancer cells (Figure 9A–D). IL‐18 is normally expressed in cancerous tissues, with a significantly higher percentage of expression in normal tissues than in tumor tissues (Figure 9E–H). Moreover, patients with higher BGN expression were predicted to have poorer survival (Figure 9I). In contrast, patients with high expression of IL‐18 showed a better prognosis (Figure 9J). High BGN expression was notably associated with tumor size, tumor invasion, and lymph node metastasis (*p* < 0.05 for all) of patients with ESCC (Table 1). For IL‐18, no significant correlation between the IL‐18 level and clinicopathological factors except lymph node metastasis (*p* < 0.001), was observed.

**TABLE 1:** Associations of BGN and IL‐18 expression with clinicopathological variables of ESCC patients from the TMA dataset

Clinicopathological variables	*n*	BGN expression		*x* ^*2*^	*p* value	IL‐18 expression		*x* ^*2*^	*p* value
		Low level	High level			Low level	High level		
Gender				1.924	0.165			3.513	0.061
Male	192	70 (36.5%)	122 (63.5%)			108 (56.3%)	84 (43.8%)		
Female	40	10 (25%)	30 (75%)			16 (40.0%)	24 (60.0%)		
Age (years)				0.837	0.360			0.595	0.440
≤66	114	36 (31.6%)	78 (68.4%)			58 (50.9%)	56 (49.1%)		
>66	118	44 (37.3%)	74 (62.7%)			66 (55.9%)	52 (44.1%)		
Tumor size (cm)				4.210	**0.040**			0.038	0.845
≤3.5	109	45 (41.3%)	64 (58.7%)			59 (54.1%)	50 (45.9%)		
>3.5	123	35 (28.5%)	88 (71.5%)			65 (52.8%)	58 (47.2%)		
Tumor location				1.088	0.581			1.710	0.425
Upper	11	5 (45.5%)	6 (54.5%)			4 (36.4%)	7 (63.6%)		
Middle	171	56 (32.7%)	115 (67.3%)			91 (53.2%)	80 (46.8%)		
Lower	50	19 (38.0%)	31 (62.0%)			29 (58.0%)	21 (42.0%)		
Histological grade				3.312	0.191			13.439	0.179
I	6	4 (66.7%)	2 (33.3%)			1 (16.7%)	5 (83.3%)		
II	169	59 (34.9%)	110 (65.1%)			91 (53.8%)	78 (46.2%)		
III	57	17 (29.8%)	40 (70.2%)			32 (56.1%)	25 (43.9%)		
Tumor invasion depth				22.483	**<0.001**			0.353	0.553
T1–T2	56	34 (60.7%)	22 (39.3%)			28 (50.0%)	28 (50.0%)		
T3–T4	176	46 (26.1%)	130 (73.9%)			96 (54.5%)	80 (45.5%)		
Lymph node metastasis				4.768	**0.029**			14.552	**<0.001**
N0	128	52 (40.6%)	76 (59.4%)			54 (42.2%)	74 (57.8%)		
N1–N3	104	28 (26.9%)	76 (73.1%)			70 (67.3%)	34 (32.7%)		

Statistically significant values are indicated in bold.

Abbreviations: BGN, biglycan; IL‐18, interleukin 18; ESCC, esophageal squamous cell carcinoma; TMA, TMA tissue microarray.

## 4 DISCUSSION

Adaptation of tumor cells to a hypoxic environment leads to increased aggressiveness and a treatment‐resistant tumor phenotype, contributing to a poor prognosis in various cancers.^3^, ^15^ Exploring mechanisms of ESCC progression can be beneficial for prognosis prediction, and some specific genomic alterations have also shown that hypoxia can induce upregulation of the expression of some genes, including CA9, VEGF, ADM, and AK3.^16^, ^17^

Since the predictive power of single indicators is limited and influenced by confounders, different hypoxia gene signatures have been reported.^18^, ^19^ A study in 2013 preliminarily explored the expression levels of 15 hypoxia‐regulated genes detected from 95 ESCC tumor paraffin samples, but it is still essentially based on individual gene expression levels rather than gene signatures and has not been further validated, so the results may still have limitations.^20^ Another study established a gene signature combining hypoxia and tumor stem cells, but our study for ESCC patients is more suitable for epidemiological characteristics in China compared to their overall analysis of esophageal cancer data.^21^ And the study scope was narrowed by consensus clustering and WGCNA methods to identify a set of genes most likely to be associated with hypoxia. No hypoxia‐related gene signature and prognostic risk prediction models have been constructed to predict hypoxia risk or to predict long‐term patient survival by exploring HRGs in esophageal squamous carcinoma. We aimed to use high‐throughput sequencing data and combine bioinformatics methods, constructing the first prognosis signature risk score model ever built containing key HRGs and the first nomogram of ESCC that encompasses both clinical attributes and the risk score.

In this study, we used publicly available databases from multiple sources for filtering rather than directly with published hypoxia genes, aiming to maximize the correlation of genes with hypoxia and screen out those genes. Futhermore, five hypoxia genes with prognostic significance were screened: SLC2A1, PGM2, SULT2B1, CA9, and BGN. The hypoxic features of these genes have been confirmed in previous studies, and genes and samples with common hypoxia exposure levels were maximally distinguished into different subgroups based on the expression levels of these genes by consensus clustering and WGCNA methods. And the DEGs between the two subgroups were matched to the screened green module genes to identify a set of genes most likely to be associated with hypoxia. The differences in the distribution of clinical features and immune infiltration between subgroups can reconfirm the accuracy and validity of the grouping. By screening and filtering in this way, we believe that both the scope of the study can be narrowed unbiasedly and the degree of hypoxia exposure can be well identified.

In this study, the first prognostic prediction model was developed based on PNPLA1, CARD18, IL‐18, SLC37A2, ADAMTS18, and FAM83C gene mRNA expression, and validated in an external validation dataset. It is suggested that the 6‐gene signature may have a cross‐platform character with good prognostic ability and generalizability in clinical applications. Our 6‐gene hypoxia signature can be used to calculate risk scores, and classifying patients into high‐ and low‐risk groups indicates different levels of hypoxia exposure. And the obtained subtypes differ in the degree of grade to help screen patients using hypoxia features before developing a treatment plan. The patients with poorly differentiation, picked higher hypoxia risk scores. It is suggested that high hypoxia scores in poorly differentiated patients are unfavorable factors for ESCC patients and predict the progression of ESCC, which may alter the outcome of treatment. These patients may respond to anti‐hypoxia therapy due to overexposure to hypoxia. The nomogram of the present study, combining age, TNM stage, and HRG risk score, yielded a favorable predictive performance. Patients with advanced ESCC who are older and have higher risk scores have lower survival rates and should be identified for more individualized treatment measures to improve long‐term survival. Age is an important prognostic indicator. The TNM stage represents a standardized benchmark to classify ESCC patients, assess prognosis, and recommend optimal treatment. The addition of the HRG risk score makes the nomogram more reliable because it correlates with the results of the training and validation sets. Compared to nomograms without risk scores, nomograms based on HRG risk scores have better performance in identification and calibration. Therefore, the current findings suggest that the established nomogram has better predictive value than the current nomograms based solely on clinicopathological features and TNM, excluding HRG risk scores. Therefore, for these specific patient groups, the gene signature of ESCC based on hypoxia exposure is clinically relevant for determining prognosis and developing individualized treatment plans.

We also investigated critical features of the tumor hypoxic environment in hopes of providing clues for clinical diagnosis and immunotherapy. Hypoxia induces changes in the proportion of specific immune cells in ESCC. It was also confirmed that esophageal squamous carcinoma cells in hypoxia could affect the tumor immune microenvironment through P53, Wnt, and hypoxia signaling pathways. However, the current evidence is insufficient to elucidate the role of immune cells. The complex interaction between tumor cells and immune cells in hypoxic environments remains to be further explored.

Two of HRGs were selected for experimental validation. Upregulation of BGN was confirmed again. BGN, a member of the family of leucine‐rich small proteoglycans (SLRPs), has been detected upregulation in esophageal,^22^, ^23^ pancreatic,^24^ gastric^25^ and was correlated with tumor progression and poor prognosis, consistent with the findings of the present study. IL‐18, the protein encoded by this gene, is a pro‐inflammatory and immunomodulatory cytokine and a member of the IL‐1 family. According to previous studies, IL‐18 activation may be a double‐edged sword that promotes tumor development and progression.^26^ In our study, it is suggested that this gene has a tumor‐suppressive effect. These findings are in line with data from studies on various other cancer types, especially ESCC.^27^ The vital role of risk signature genes identified in this study has been previously reported in cancer except for SLC37A2. PNPLA1 is associated with lipid metabolism, and its nonsense mutation has been identified in human cervical cancer HeLa cells.^28^ The expression level of CARD18 was found to be significantly higher in gastric adenocarcinoma tissues than in paracancerous tissues, and the mRNA and protein levels of CARD18 were significantly downregulated after apoptotic treatment.^29^ It can be used as a prognostic indicator and therapeutic target protein for gastric cancer. ADAMTS18 played a complex role in tumor development. As a tumor suppressor gene, its methylation could inhibit the migration and invasion of breast cancer cells.^30^ It is downregulated by methylation in several cancer types such as lung cancer^31^ and clear cell carcinoma^32^ as a potential prognostic biomarker. These results are consistent with the current findings that ADAMTS18 was identified as a gene associated with a good prognosis in esophageal cancer. Nevertheless, ADAMTS18 is highly expressed in stomach adenocarcinoma^33^ and therefore may serve as a potential indicator of poor prognosis. High expression of FAM83C‐AS1 indicated poor prognosis in colon cancer.^34^

The following are limitations of this study: First, from the perspective of data sources, potential selection bias could not be ruled out due to data obtained from the TCGA and GEO databases. Most of these patients were white, which may result in inconsistent RNA‐seq results and poor reproducibility of other races. Second, certain limitations and preferences could not be excluded as these were retrospective analyses. Some confounders, such as therapeutic methods and lifestyle factors, influencing the prognosis were unavailable from the GEO and TCGA databases. Third, Further in vitro and in vivo studies are needed to investigate the mechanisms of the critical genes.

In conclusion, our study identified a 6‐gene signature and a prognostic nomogram incorporating the gene signature and clinical prognostic factors associated with tumor prognosis. The constituents of the gene signature may serve as potential prognostic predictors and provide therapeutic targets with future clinical applications for ESCC. The prognostic nomogram may reliably facilitate individualized treatment and medical decision‐making.

## AUTHOR CONTRIBUTION

Conception and design: Xiao Wanyi, Tang Peng, Yu Zhentao, and Zhang Hongdian; Administrative support: Zhao Gang, Gong Lei, Yu Zhentao, and Zhang Hongdian; Provision of study materials or patients: Tang Peng, Sui Zhilin and Han Youming; Collection and assembly of data: Sui Zhilin, Wu Xianxian, and Han Youming; Data analysis and interpretation: Xiao Wanyi, Tang Peng and Zhang Hongdian; Manuscript writing: Xiao Wanyi; Manuscript revision: Xiao Wanyi, Yang Yueyang, and Zhu Ningning. Final approval of manuscript: All authors.

## CONFLICT OF INTEREST

No potential conflict of interest is disclosed.

## ETHICS APPROVAL AND CONSENT TO PARTICIPATE

This study was performed with the approval of the Research Ethics Committee of Tianjin Medical University Cancer Institute and Hospital.

## Supporting information


FigureS1‐S6

TableS1‐S2

## Data Availability

Deidentified data and related documents will be made available upon request.

## References

[1] He Z , Ke Y . Precision screening for esophageal squamous cell carcinoma in China. Chin J Cancer Res. 2020;32(6):673‐682. 33446991 10.21147/j.issn.1000-9604.2020.06.01 PMC7797228

[2] Zhang J , Wang JL , Zhang CY , Ma YF , Zhao R , Wang YY . The prognostic role of FZD6 in esophageal squamous cell carcinoma patients. Clin Transl Oncol. 2020;22(7):1172‐1179. 31748958 10.1007/s12094-019-02243-3

[3] Jing X , Yang F , Shao C , et al. Role of hypoxia in cancer therapy by regulating the tumor microenvironment. Mol Cancer. 2019;18(1):157. 31711497 10.1186/s12943-019-1089-9 PMC6844052

[4] Chen F , Chu L , Li J , et al. Hypoxia induced changes in miRNAs and their target mRNAs in extracellular vesicles of esophageal squamous cancer cells. Thorac Cancer. 2020;11(3):570‐580. 31922357 10.1111/1759-7714.13295 PMC7049507

[5] Wang Y , Xie Z , Lu H . Significance of halofuginone in esophageal squamous carcinoma cell apoptosis through HIF‐1alpha‐FOXO3a pathway. Life Sci. 2020;257:118104. 32679143 10.1016/j.lfs.2020.118104

[6] Macedo‐Silva C , Miranda‐Goncalves V , Lameirinhas A , et al. JmjC‐KDMs KDM3A and KDM6B modulate radioresistance under hypoxic conditions in esophageal squamous cell carcinoma. Cell Death Dis. 2020;11(12):1068. 33318475 10.1038/s41419-020-03279-y PMC7736883

[7] Vaupel P , Mayer A . Hypoxia in cancer: significance and impact on clinical outcome. Cancer Metastasis Rev. 2007;26(2):225‐239. 17440684 10.1007/s10555-007-9055-1

[8] Muz B , de la Puente P , Azab F , Azab AK . The role of hypoxia in cancer progression, angiogenesis, metastasis, and resistance to therapy. Hypoxia (Auckl). 2015;3:83‐92. 27774485 10.2147/HP.S93413 PMC5045092

[9] Mao Y , Wang Y , Dong L , et al. Hypoxic exosomes facilitate angiogenesis and metastasis in esophageal squamous cell carcinoma through altering the phenotype and transcriptome of endothelial cells. J Exp Clin Cancer Res. 2019;38(1):389. 31488217 10.1186/s13046-019-1384-8 PMC6727585

[10] Liu M , An H , Zhang Y , et al. Molecular analysis of Chinese oesophageal squamous cell carcinoma identifies novel subtypes associated with distinct clinical outcomes. EBioMedicine. 2020;57:102831. 32580137 10.1016/j.ebiom.2020.102831 PMC7317223

[11] van Uden P , Kenneth NS , Rocha S . Regulation of hypoxia‐inducible factor‐1alpha by NF‐kappaB. Biochem J. 2008;412(3):477‐484. 18393939 10.1042/BJ20080476 PMC2474706

[12] Yuan H , Yan M , Zhang G , et al. CancerSEA: a cancer single‐cell state atlas. Nucleic Acids Res. 2019;47(D1):D900‐D908. 30329142 10.1093/nar/gky939 PMC6324047

[13] Liberzon A , Birger C , Thorvaldsdottir H , Ghandi M , Mesirov JP , Tamayo P . The molecular signatures database (MSigDB) hallmark gene set collection. Cell Syst. 2015;1(6):417‐425. 26771021 10.1016/j.cels.2015.12.004 PMC4707969

[14] Langfelder P , Horvath S . WGCNA: an R package for weighted correlation network analysis. BMC Bioinformatics. 2008;9:559. 19114008 10.1186/1471-2105-9-559 PMC2631488

[15] Akanji MA , Rotimi D , Adeyemi OS . Hypoxia‐inducible factors as an alternative source of treatment strategy for cancer. Oxid Med Cell Longev. 2019;2019:8547846‐8547810. 31485300 10.1155/2019/8547846 PMC6710762

[16] Harris BH , Barberis A , West CM , Buffa FM . Gene expression signatures as biomarkers of tumour hypoxia. Clin Oncol (R Coll Radiol). 2015;27(10):547‐560. 26282471 10.1016/j.clon.2015.07.004

[17] Yang L , West CM . Hypoxia gene expression signatures as predictive biomarkers for personalising radiotherapy. Br J Radiol. 2019;92(1093):20180036. 29513038 10.1259/bjr.20180036 PMC6435086

[18] Shou Y , Yang L , Yang Y , Zhu X , Li F , Xu J . Identification of signatures of prognosis prediction for melanoma using a hypoxia score. Front Genet. 2020;11:570530. 33133157 10.3389/fgene.2020.570530 PMC7550673

[19] Yang Y , Li Y , Qi R , Zhang L . Constructe a novel 5 hypoxia genes signature for cervical cancer. Cancer Cell Int. 2021;21(1):345. 34217310 10.1186/s12935-021-02050-3 PMC8254931

[20] Winther M , Alsner J , Tramm T , Nordsmark M . Hypoxia‐regulated gene expression and prognosis in loco‐regional gastroesophageal cancer. Acta Oncol. 2013;52(7):1327‐1335. 23957682 10.3109/0284186X.2013.818247

[21] Tang K , Cheng Y , Li Q . Construction and verification of a hypoxia‐stemness‐based gene signature for risk stratification in esophageal cancer. Med Sci Monit. 2021;27:e934359‐e. 34716287 10.12659/MSM.934359 PMC8565098

[22] Zhu YH , Yang F , Zhang SS , Zeng TT , Xie X , Guan XY . High expression of biglycan is associated with poor prognosis in patients with esophageal squamous cell carcinoma. Int J Clin Exp Pathol. 2013;6(11):2497‐2505. 24228112 PMC3816819

[23] Tang L , Chen Y , Peng X , et al. Identification and validation of potential pathogenic genes and prognostic markers in ESCC by integrated bioinformatics analysis. Front Genet. 2020;11:521004. 33362844 10.3389/fgene.2020.521004 PMC7758294

[24] Yang L , Cui R , Li Y , Liang K , Ni M , Gu Y . Hypoxia‐induced TGFBI as a serum biomarker for laboratory diagnosis and prognosis in patients with pancreatic ductal adenocarcinoma. Lab Med. 2020;51(4):352‐361. 31626700 10.1093/labmed/lmz063

[25] Liu Z , Liu S , Guo J , et al. Identification and analysis of key genes driving gastric cancer through bioinformatics. Genet Test Mol Biomarkers. 2021;25(1):1‐11. 33470887 10.1089/gtmb.2020.0126

[26] Saetang J , Chonpathompikunlert P , Sretrirutchai S , et al. Anti‐cancer effect of engineered recombinant interleukin 18. Adv Clin Exp Med. 2020;29(10):1135‐1143. 33031647 10.17219/acem/126298

[27] Tsuboi K , Miyazaki T , Nakajima M , et al. Serum interleukin‐12 and interleukin‐18 levels as a tumor marker in patients with esophageal carcinoma. Cancer Lett. 2004;205(2):207‐214. 15036653 10.1016/j.canlet.2003.10.010

[28] Chang PA , Sun YJ , Huang FF , et al. Identification of human patatin‐like phospholipase domain‐containing protein 1 and a mutant in human cervical cancer HeLa cells. Mol Biol Rep. 2013;40(10):5597‐5605. 24057234 10.1007/s11033-013-2661-9

[29] Tan C , Liu S , Xiang Z . The expression of CARD18 in apoptin‐transfected gastric cancer cells and gastric adenocarcinoma tissues. Xi Bao Yu Fen Zi Mian Yi Xue Za Zhi. 2013;29(8):858‐861. 23948415

[30] Xu H , Xiao Q , Fan Y , et al. Epigenetic silencing of ADAMTS18 promotes cell migration and invasion of breast cancer through AKT and NF‐κB signaling. Cancer Med. 2017;6(6):1399‐1408. 28503860 10.1002/cam4.1076 PMC5463072

[31] Zhang Y , Xu H , Mu J , et al. Inactivation of ADAMTS18 by aberrant promoter hypermethylation contribute to lung cancer progression. J Cell Physiol. 2019;234(5):6965‐6975. 30417422 10.1002/jcp.27439

[32] Xu B , Zhang L , Luo C , et al. Hypermethylation of the 16q23.1 tumor suppressor gene ADAMTS18 in clear cell renal cell carcinoma. Int J Mol Sci. 2015;16(1):1051‐1065. 25569086 10.3390/ijms16011051 PMC4307290

[33] Jiang K , Li L , Xie Y , Xie D , Xiao Q . High ADAMTS18 expression is associated with poor prognosis in stomach adenocarcinoma. Oncol Lett. 2020;20(5):211. 32963617 10.3892/ol.2020.12074 PMC7491029

[34] Xue W , Wang F , Han P , et al. The oncogenic role of LncRNA FAM83C‐AS1 in colorectal cancer development by epigenetically inhibits SEMA3F via stabilizing EZH2. Aging (Albany NY). 2020;12(20):20396‐20412. 33109776 10.18632/aging.103835 PMC7655168

