# Supplementary material for: Establishment of a risk model by integrating hypoxia genes in predicting prognosis of esophageal squamous cell carcinoma
Source: Cancer Med. 2022 Jul 4;12(2):2117–33. doi: 10.1002/cam4.5002 (PMC9883439; doi:10.1002/cam4.5002)
Supplement: Supplementary file 1 — FigureS1‐S6 [file CAM4-12-2117-s001.docx]

**Supporting Information**

**Figure S1**


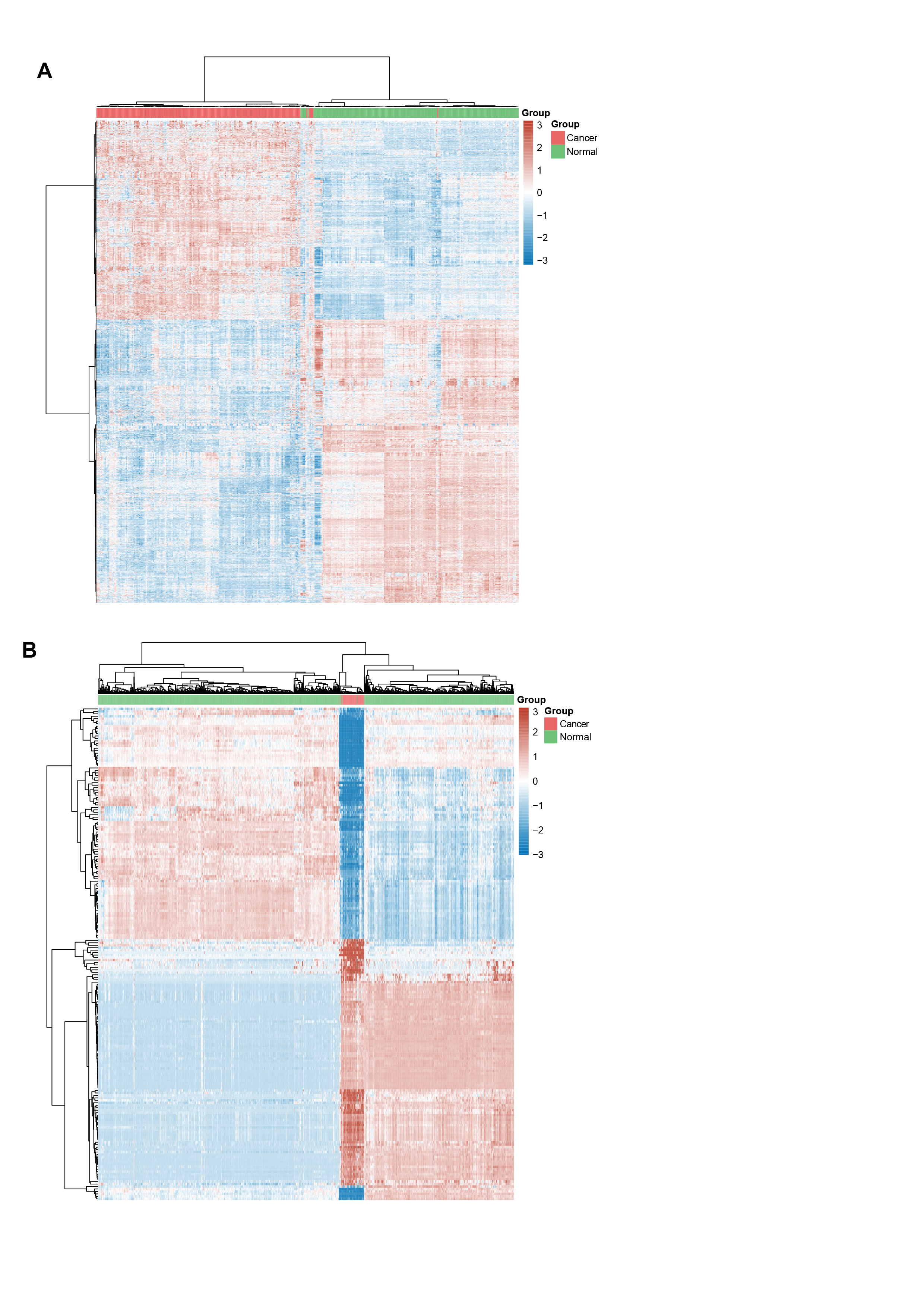


Figure. S1. Heat maps were used display the differentially expressed genes of GEO dataset (A) and TCGA (B) dataset.

**Figure S2**


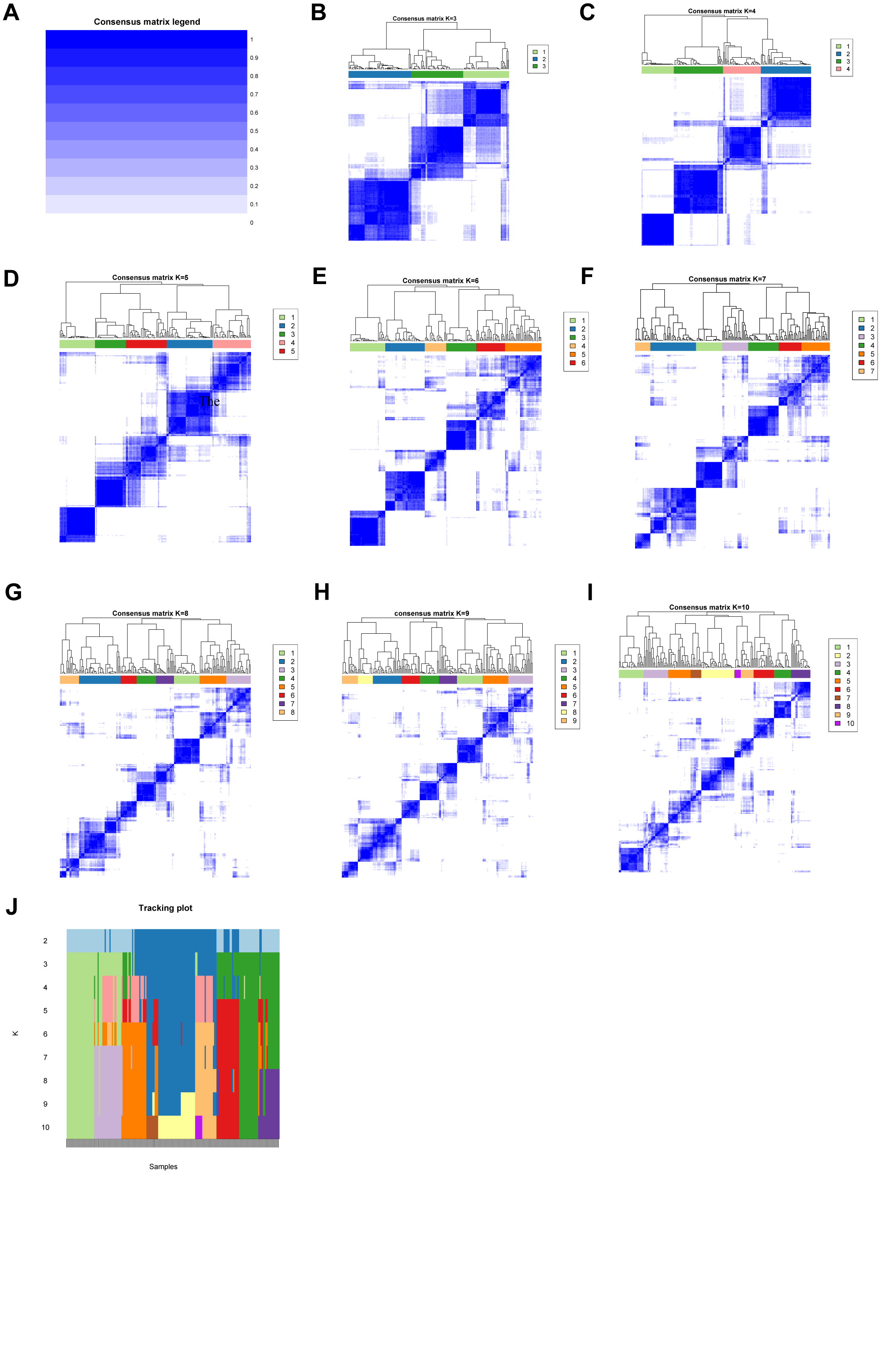
Figure. S2. Detailed results of the consensus clustering analysis.

**Figure S3**


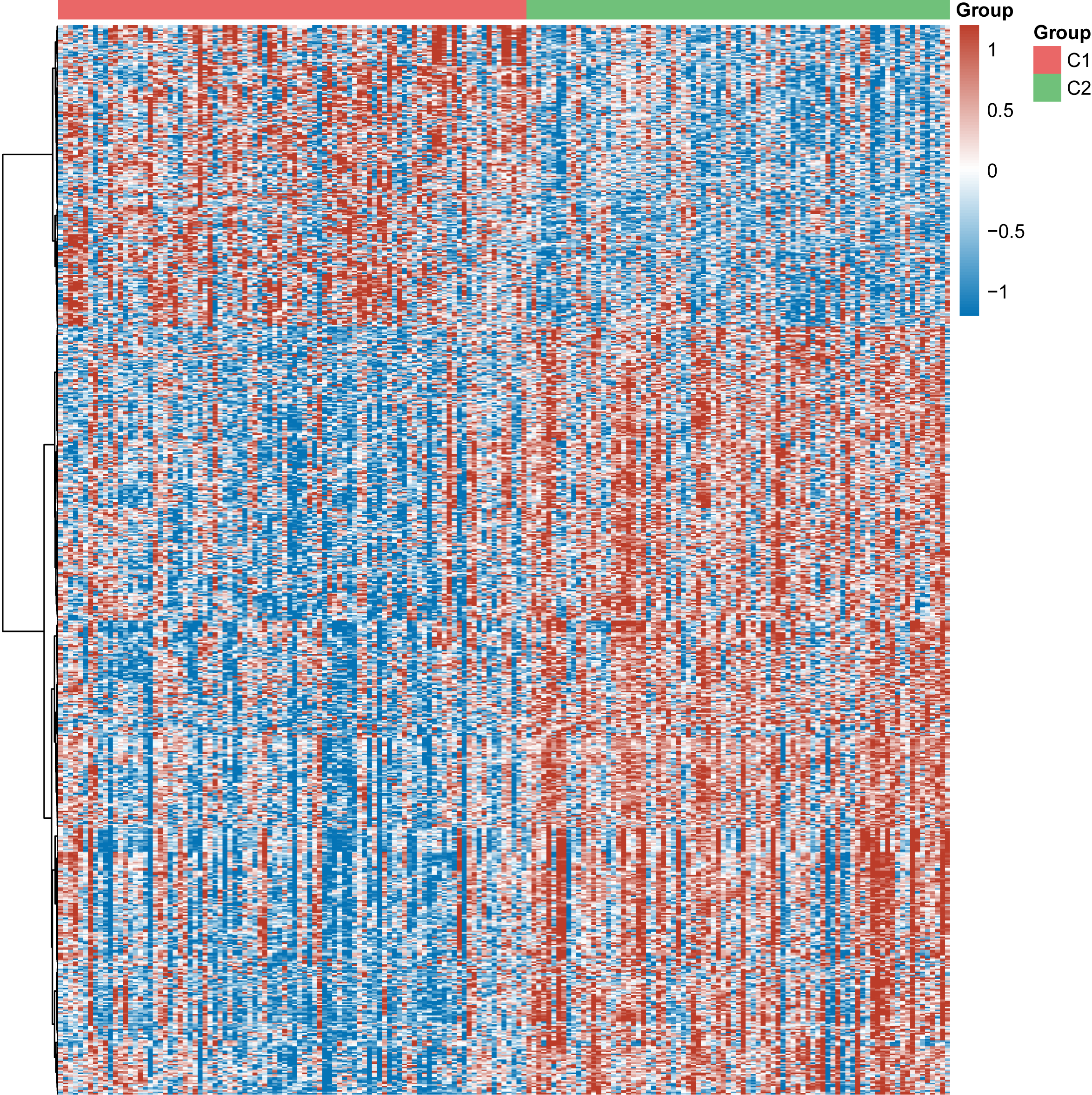
Figure. S3. A heat map was used display the gene expression profiles of the samples sorted by cluster.

**Figure S4**


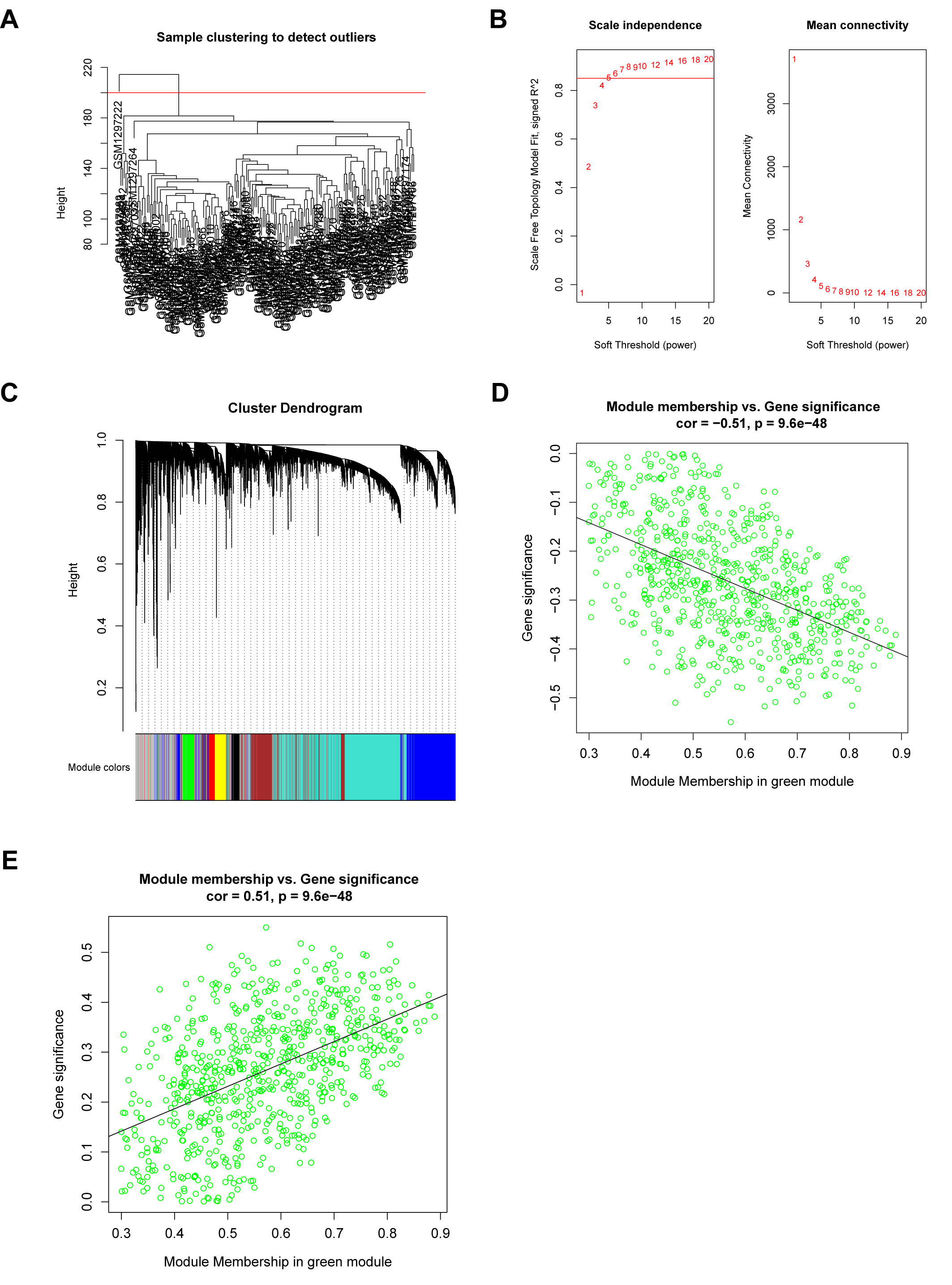
Figure. S4. Detailed results of the weighted gene co-expression network analysis (A-C). The results of module-feature relationship analysis between green module and consensus subgroup C1 (D) and consensus subgroup C2 (E), suggesting that the module is suitable for identifying the hub genes associated with C1/C2.

**Figure S5**


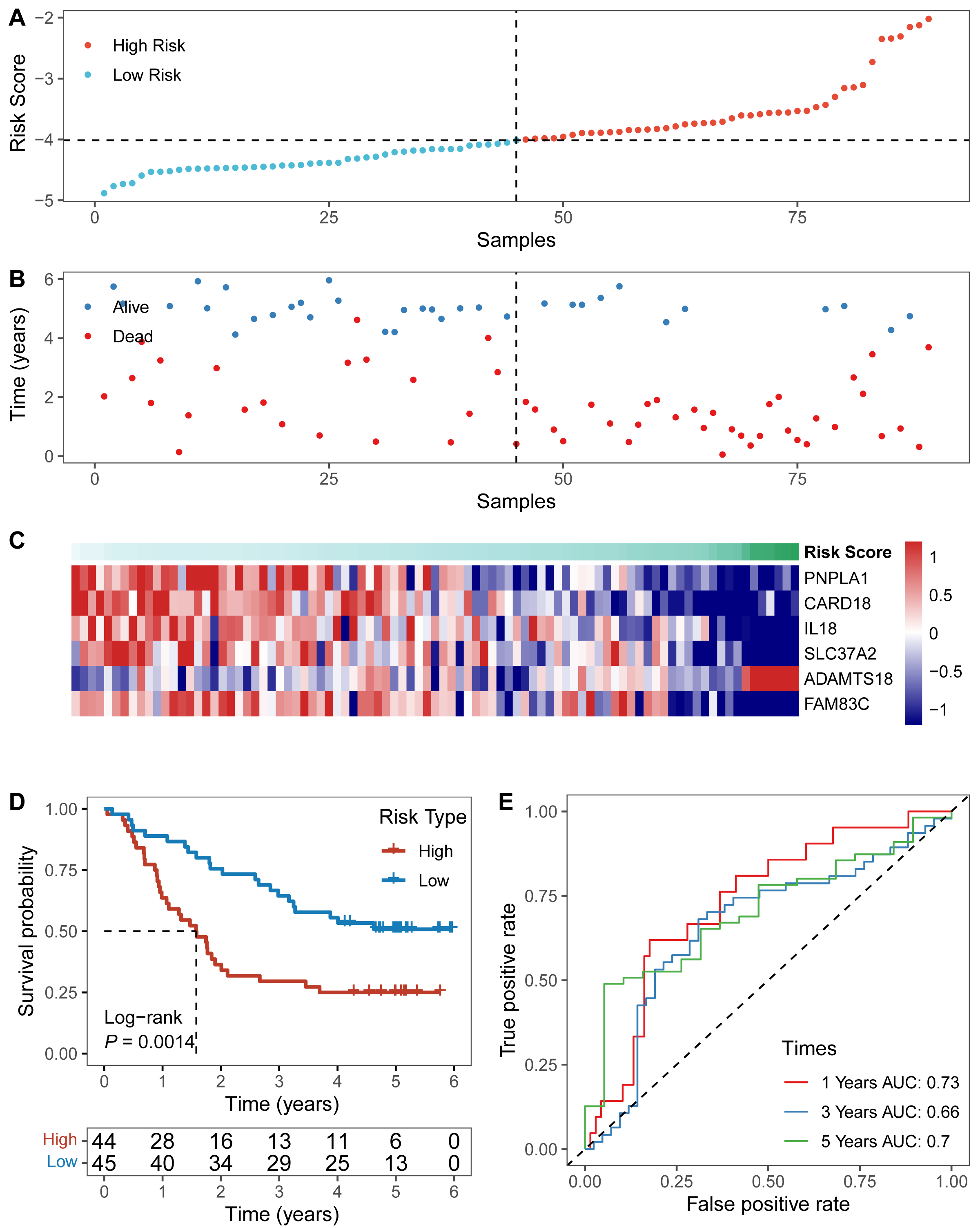
Figure. S5. Validating the prognostic hypoxia gene features in the remaining half GSE53625 dataset. The samples were assigned a risk score and ordered to determine whether the expression level (A) and survival time (B) varied systematically with the risk score. (C) Expression levels of the 6 HRGs based on risk scores. (D) Survival curve distribution of the risk score. (E) ROC curves and AUCs of risk score classifications.

Abbreviations: HRGs, hypoxia related genes.

**Figure S6**


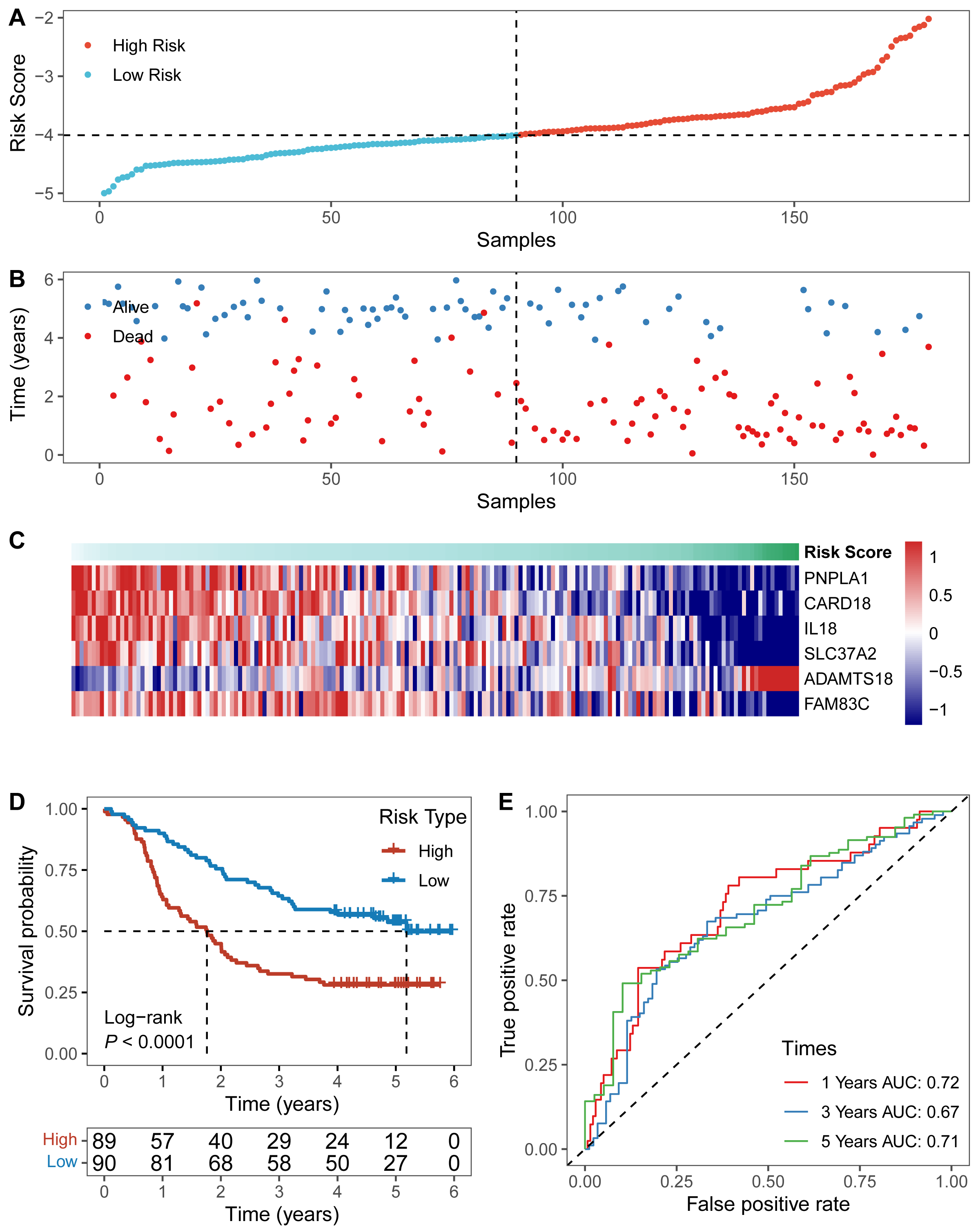


Figure. S6. Validating the prognostic hypoxia gene features in the entire GSE53625 dataset. The samples were assigned a risk score and ordered to determine whether the expression level (A) and survival time (B) varied systematically with the risk score. (C) Expression levels of the 6 HRGs based on risk scores. (D) Survival curve distribution of the risk score. (E) ROC curves and AUCs of risk score classifications.

Abbreviations: HRGs, hypoxia related genes.
