# Supplementary material for: Establishment of a risk model by integrating hypoxia genes in predicting prognosis of esophageal squamous cell carcinoma
Source: Cancer Med. 2022 Jul 4;12(2):2117–33. doi: 10.1002/cam4.5002 (PMC9883439; doi:10.1002/cam4.5002)
Supplement: Supplementary file 2 — TableS1‐S2 [file CAM4-12-2117-s002.docx]

**Table S1** 30 consistently differentially expressed genes in the GEO training set and TCGA validation set.

| Genes | P.Value | HR | Lower 95 CI | Upper 95 CI |
| --- | --- | --- | --- | --- |
| SLC2A1 | 0.0062227 | 0.7469831 | 0.6061068 | 0.920603 |
| P4HA1 | 0.9303723 | 1.0093968 | 0.8183669 | 1.2450185 |
| ERO1A | 0.0905733 | 0.8374722 | 0.6819642 | 1.0284405 |
| SERPINE1 | 0.2464891 | 1.1223675 | 0.9233081 | 1.3643427 |
| LOX | 0.7071523 | 0.9721177 | 0.8387784 | 1.1266536 |
| IGFBP3 | 0.4549767 | 1.0762695 | 0.8875306 | 1.3051449 |
| PYGM | 0.1712466 | 1.1946856 | 0.9259501 | 1.5414153 |
| CHST2 | 0.0610837 | 0.8922443 | 0.7918911 | 1.0053149 |
| TMEM45A | 0.5598608 | 1.0789334 | 0.8357579 | 1.3928642 |
| PLAUR | 0.5458942 | 0.9296254 | 0.733593 | 1.1780419 |
| NOCT | 0.3480825 | 0.9178192 | 0.7672975 | 1.0978689 |
| KDELR3 | 0.1226575 | 1.1818294 | 0.9559523 | 1.4610778 |
| TNFAIP3 | 0.9512049 | 0.9921363 | 0.7704686 | 1.2775788 |
| BGN | 0.0463427 | 1.310797 | 1.0043966 | 1.7106675 |
| SRPX | 0.1458652 | 1.1087697 | 0.9647354 | 1.2743082 |
| TGFBI | 0.4087764 | 1.1181843 | 0.857839 | 1.4575416 |
| AKAP12 | 0.0977147 | 1.2006185 | 0.967001 | 1.4906757 |
| GPC1 | 0.5698922 | 0.9531898 | 0.8079072 | 1.1245979 |
| SELENBP1 | 0.3432446 | 1.0948296 | 0.9077771 | 1.3204254 |
| SULT2B1 | 0.0035315 | 0.8322966 | 0.7357264 | 0.9415424 |
| S100A10 | 0.8408899 | 1.0330441 | 0.752103 | 1.418928 |
| CLU | 0.6987979 | 1.0333025 | 0.8753099 | 1.2198127 |
| TNC | 0.7169525 | 0.9714505 | 0.8306408 | 1.1361301 |
| GADD45B | 0.2449866 | 1.1703579 | 0.8977289 | 1.525781 |
| SLCO4A1 | 0.526913 | 0.9257424 | 0.7289341 | 1.1756879 |
| CA9 | 0.0315508 | 0.8970025 | 0.8123831 | 0.9904359 |
| FOSB | 0.2230782 | 0.8755531 | 0.7070248 | 1.0842521 |
| PDZK1IP1 | 0.0511284 | 0.8873485 | 0.7869261 | 1.0005862 |
| LAMB3 | 0.2223763 | 1.1750741 | 0.9068283 | 1.5226687 |
| PGM2 | 0.0102265 | 0.6780708 | 0.5040847 | 0.9121087 |

GEO, Gene Expression Omnibus; TCGA, The Cancer Genome Atlas; HR, hazard ratio; CI, coincidence interval.

**Table S2** Associations of the immune‐related score and the risk score between ESCC patients in the high and low‐risk groups.

| Sample | Risk Type | Cluster | Stromal Score | Immune Score | Estimate Score | CD274 |
| --- | --- | --- | --- | --- | --- | --- |
| GSM1296956 | High | 1 | 1312.1857 | 2315.8268 | 3628.0125 | 9.977063 |
| GSM1296958 | Low | 2 | -315.26 | 598.88195 | 283.62195 | 7.999674 |
| GSM1296960 | High | 1 | 430.61332 | 995.66677 | 1426.2801 | 9.649608 |
| GSM1296962 | High | 1 | 651.57592 | 379.41175 | 1030.9877 | 6.6095004 |
| GSM1296964 | High | 1 | 117.07822 | 766.25671 | 883.33493 | 7.9468665 |
| GSM1296966 | High | 1 | 493.47903 | 1934.5083 | 2427.9873 | 9.077539 |
| GSM1296968 | High | 2 | 754.16484 | 1057.2861 | 1811.451 | 8.956123 |
| GSM1296970 | High | 2 | 760.01314 | 1110.9434 | 1870.9565 | 8.012805 |
| GSM1296972 | High | 1 | 569.76007 | 1472.3209 | 2042.0809 | 8.556532 |
| GSM1296974 | Low | 2 | 1367.8845 | 1928.5183 | 3296.4028 | 10.007777 |
| GSM1296976 | Low | 2 | 151.25467 | 638.82156 | 790.07623 | 7.26219 |
| GSM1296978 | Low | 2 | 356.02507 | 1058.6118 | 1414.6369 | 10.985563 |
| GSM1296980 | Low | 2 | -125.0329 | 863.43518 | 738.40228 | 8.272133 |
| GSM1296982 | High | 1 | 771.39248 | 1498.9766 | 2270.3691 | 8.396713 |
| GSM1296984 | Low | 1 | 1266.0397 | 1534.3255 | 2800.3652 | 8.693287 |
| GSM1296986 | Low | 1 | 910.95879 | 1271.7208 | 2182.6796 | 7.8418703 |
| GSM1296988 | Low | 2 | 391.5885 | 663.20433 | 1054.7928 | 9.882932 |
| GSM1296990 | Low | 2 | 1172.2167 | 866.03131 | 2038.248 | 8.295491 |
| GSM1296992 | Low | 1 | 867.00673 | 1431.7559 | 2298.7626 | 9.009955 |
| GSM1296994 | Low | 2 | -0.384436 | 626.38686 | 626.00242 | 8.8393545 |
| GSM1296996 | High | 1 | 419.303 | 776.07118 | 1195.3742 | 10.368077 |
| GSM1296998 | Low | 2 | 654.02881 | 800.3261 | 1454.3549 | 7.812298 |
| GSM1297000 | High | 1 | 394.28466 | 1024.1671 | 1418.4517 | 7.48386 |
| GSM1297002 | Low | 2 | 321.5603 | 778.59512 | 1100.1554 | 7.2554135 |
| GSM1297004 | High | 2 | 407.88052 | 1231.023 | 1638.9036 | 9.569848 |
| GSM1297006 | Low | 2 | 244.6802 | 686.84186 | 931.52206 | 6.3058004 |
| GSM1297008 | Low | 2 | 821.25937 | 814.33335 | 1635.5927 | 6.8889575 |
| GSM1297010 | Low | 1 | -15.22556 | 538.17413 | 522.94857 | 7.9204392 |
| GSM1297012 | High | 1 | 539.40716 | 1077.5447 | 1616.9519 | 8.719577 |
| GSM1297014 | Low | 2 | 1005.271 | 1385.2283 | 2390.4993 | 8.651842 |
| GSM1297016 | High | 1 | 1506.176 | 1375.5159 | 2881.6919 | 8.20136 |
| GSM1297018 | High | 1 | -65.38878 | 165.60467 | 100.21589 | 9.021519 |
| GSM1297020 | High | 1 | 1567.0493 | 1814.6188 | 3381.668 | 8.9035225 |
| GSM1297022 | High | 1 | -36.6916 | 803.73189 | 767.04029 | 6.8095765 |
| GSM1297024 | High | 1 | 861.19268 | 855.94335 | 1717.136 | 7.2601633 |
| GSM1297026 | Low | 1 | 1159.3231 | 1278.2779 | 2437.601 | 7.77319 |
| GSM1297028 | Low | 2 | 1046.3409 | 922.17145 | 1968.5124 | 10.24087 |
| GSM1297030 | Low | 2 | 511.15458 | 848.41079 | 1359.5654 | 9.014844 |
| GSM1297032 | High | 1 | 460.37881 | 708.39274 | 1168.7716 | 5.4452453 |
| GSM1297034 | High | 1 | 445.24476 | 304.61306 | 749.85782 | 5.8199687 |
| GSM1297036 | Low | 2 | 401.5746 | 83.712665 | 485.28726 | 6.681517 |
| GSM1297038 | High | 1 | 306.85374 | 1203.0698 | 1509.9235 | 8.114721 |
| GSM1297040 | High | 1 | 767.7774 | 692.95739 | 1460.7348 | 8.943167 |
| GSM1297042 | High | 1 | 199.04169 | 321.34527 | 520.38695 | 6.597915 |
| GSM1297044 | High | 1 | 886.95053 | 1231.8642 | 2118.8147 | 7.914528 |
| GSM1297046 | Low | 2 | 413.38734 | 892.56576 | 1305.9531 | 8.717353 |
| GSM1297048 | High | 1 | 1049.7721 | 1870.2726 | 2920.0447 | 8.548778 |
| GSM1297050 | High | 1 | 1067.299 | 1257.1828 | 2324.4818 | 8.230229 |
| GSM1297052 | High | 2 | 1264.5081 | 1027.5613 | 2292.0694 | 8.495483 |
| GSM1297054 | Low | 1 | 258.31922 | 615.70985 | 874.02907 | 7.801359 |
| GSM1297056 | High | 1 | 1495.1106 | 1401.5812 | 2896.6918 | 8.278519 |
| GSM1297058 | Low | 2 | 412.60129 | 189.5333 | 602.13459 | 7.8022184 |
| GSM1297060 | Low | 1 | 129.42892 | 167.53754 | 296.96646 | 7.6131988 |
| GSM1297062 | Low | 1 | 531.62699 | 398.51036 | 930.13735 | 8.706822 |
| GSM1297064 | High | 1 | 1125.434 | 1869.9467 | 2995.3807 | 8.368032 |
| GSM1297066 | Low | 2 | 227.74274 | 913.50308 | 1141.2458 | 7.3102856 |
| GSM1297068 | Low | 2 | 1047.6463 | 951.60579 | 1999.2521 | 6.9871554 |
| GSM1297070 | Low | 2 | 1029.4426 | 759.55156 | 1788.9942 | 7.3856936 |
| GSM1297072 | Low | 2 | -250.2108 | 240.21891 | -9.991928 | 7.9800787 |
| GSM1297074 | High | 1 | 566.0979 | 661.89514 | 1227.993 | 6.104688 |
| GSM1297076 | Low | 2 | 344.64533 | 246.91631 | 591.56164 | 6.677686 |
| GSM1297078 | High | 2 | 629.04256 | 867.23044 | 1496.273 | 6.8331175 |
| GSM1297080 | Low | 2 | 1072.4396 | 776.18305 | 1848.6226 | 7.05477 |
| GSM1297082 | Low | 2 | 901.41784 | 1970.4634 | 2871.8813 | 8.339965 |
| GSM1297084 | Low | 2 | 481.71901 | 1051.8736 | 1533.5926 | 9.473089 |
| GSM1297086 | High | 1 | 390.221 | 1063.7586 | 1453.9796 | 7.684279 |
| GSM1297088 | Low | 2 | -125.3887 | 951.30859 | 825.91987 | 9.024005 |
| GSM1297090 | Low | 1 | 449.28136 | 979.97704 | 1429.2584 | 8.47708 |
| GSM1297092 | High | 1 | 573.50066 | 814.83029 | 1388.3309 | 6.465546 |
| GSM1297094 | High | 1 | 1378.704 | 1523.9727 | 2902.6767 | 7.9900136 |
| GSM1297096 | High | 2 | 327.90261 | 510.41525 | 838.31786 | 9.198437 |
| GSM1297098 | High | 1 | 621.30226 | 1318.4238 | 1939.7261 | 9.6537 |
| GSM1297100 | High | 1 | 755.2752 | 1192.1905 | 1947.4657 | 7.1673245 |
| GSM1297102 | Low | 2 | 829.04157 | 1304.1361 | 2133.1777 | 8.655793 |
| GSM1297104 | High | 2 | 664.34868 | 732.97675 | 1397.3254 | 7.2981477 |
| GSM1297106 | High | 1 | 673.54651 | 1174.3138 | 1847.8603 | 7.765467 |
| GSM1297108 | Low | 2 | -156.9364 | 847.39144 | 690.45499 | 10.385633 |
| GSM1297110 | Low | 2 | 476.70719 | 963.53966 | 1440.2469 | 7.3476925 |
| GSM1297112 | Low | 2 | 224.5098 | 675.60391 | 900.11371 | 7.4858627 |
| GSM1297114 | High | 2 | 740.21485 | 1402.4035 | 2142.6184 | 10.157433 |
| GSM1297116 | Low | 1 | 528.16028 | 1274.4591 | 1802.6194 | 8.75019 |
| GSM1297118 | Low | 2 | 1044.8505 | 1445.7005 | 2490.551 | 8.425886 |
| GSM1297120 | Low | 2 | -658.7144 | -231.7092 | -890.4237 | 7.151233 |
| GSM1297122 | High | 1 | 531.75057 | 682.41945 | 1214.17 | 7.284304 |
| GSM1297124 | Low | 2 | 1062.4768 | 1143.0728 | 2205.5496 | 8.221296 |
| GSM1297126 | Low | 1 | -260.4659 | -206.5825 | -467.0485 | 8.893638 |
| GSM1297128 | Low | 1 | 360.84796 | 1514.54 | 1875.3879 | 7.706537 |
| GSM1297130 | Low | 2 | -205.1374 | 216.45671 | 11.319306 | 7.435209 |
| GSM1297132 | High | 1 | 94.267877 | 895.70079 | 989.96866 | 5.821306 |
| GSM1297134 | High | 2 | 176.96745 | 224.67568 | 401.64313 | 7.1061306 |
| GSM1297136 | High | 2 | -160.8487 | -75.8459 | -236.6946 | 7.4911733 |
| GSM1297138 | High | 1 | -284.783 | -67.42936 | -352.2124 | 5.399987 |
| GSM1297140 | High | 1 | -710.1777 | -339.4589 | -1049.637 | 8.534845 |
| GSM1297142 | High | 1 | 921.51789 | 1434.5176 | 2356.0355 | 7.8468657 |
| GSM1297144 | Low | 1 | -513.3957 | 710.58921 | 197.19347 | 9.285962 |
| GSM1297146 | High | 1 | -769.7979 | 666.27056 | -103.5273 | 7.8069715 |
| GSM1297148 | High | 1 | 204.43554 | 989.47689 | 1193.9124 | 8.84647 |
| GSM1297150 | High | 1 | 1110.6706 | 1942.6481 | 3053.3187 | 8.267776 |
| GSM1297152 | Low | 2 | 232.72584 | 236.70282 | 469.42866 | 6.397453 |
| GSM1297154 | High | 1 | -640.2145 | 152.79156 | -487.4229 | 7.0641403 |
| GSM1297156 | High | 1 | -383.0316 | -239.1958 | -622.2274 | 7.575291 |
| GSM1297158 | High | 1 | 587.26922 | 144.99694 | 732.26615 | 6.219926 |
| GSM1297160 | High | 1 | 235.44742 | 663.84306 | 899.29048 | 10.85433 |
| GSM1297162 | Low | 1 | -133.6198 | 118.68718 | -14.93263 | 6.2725368 |
| GSM1297164 | Low | 2 | 1071.1218 | 1546.7797 | 2617.9016 | 9.516679 |
| GSM1297166 | Low | 2 | 616.19718 | 1199.705 | 1815.9022 | 7.696523 |
| GSM1297168 | High | 1 | 717.43421 | 920.46636 | 1637.9006 | 10.139408 |
| GSM1297170 | High | 2 | 652.99103 | 831.77589 | 1484.7669 | 7.2616754 |
| GSM1297172 | Low | 2 | 240.22309 | 1016.6055 | 1256.8286 | 7.7812033 |
| GSM1297174 | High | 1 | 180.49432 | 412.62809 | 593.12242 | 7.3078756 |
| GSM1297176 | High | 1 | 1362.9573 | 1831.8582 | 3194.8155 | 9.070656 |
| GSM1297178 | High | 1 | 1252.6705 | 1917.856 | 3170.5265 | 7.6040463 |
| GSM1297180 | Low | 2 | 322.98591 | 892.92979 | 1215.9157 | 7.630212 |
| GSM1297182 | Low | 2 | 691.88589 | 1017.6937 | 1709.5796 | 7.7717223 |
| GSM1297184 | High | 2 | 1021.1543 | 1282.0989 | 2303.2532 | 9.65104 |
| GSM1297186 | Low | 1 | 1400.3313 | 1991.5684 | 3391.8997 | 8.187296 |
| GSM1297188 | Low | 2 | 286.02856 | 749.55606 | 1035.5846 | 7.750438 |
| GSM1297190 | Low | 2 | -166.8632 | 722.59007 | 555.7269 | 7.7908287 |
| GSM1297192 | Low | 2 | 679.84307 | 818.83723 | 1498.6803 | 7.6839685 |
| GSM1297194 | High | 1 | 1215.1834 | 1513.6458 | 2728.8292 | 7.5337577 |
| GSM1297196 | High | 2 | 847.27464 | 1904.497 | 2751.7716 | 8.512612 |
| GSM1297198 | Low | 2 | 388.93393 | 771.14638 | 1160.0803 | 8.548593 |
| GSM1297200 | Low | 1 | 745.3606 | 1479.6369 | 2224.9975 | 8.222227 |
| GSM1297202 | High | 2 | 309.33588 | 881.26334 | 1190.5992 | 9.680141 |
| GSM1297204 | Low | 2 | 1069.1664 | 1555.4194 | 2624.5858 | 8.414146 |
| GSM1297206 | High | 1 | 781.16067 | 1751.2478 | 2532.4085 | 8.234506 |
| GSM1297208 | Low | 2 | 6.3294804 | 1138.5915 | 1144.921 | 9.313619 |
| GSM1297210 | High | 2 | -49.18983 | 773.53265 | 724.34282 | 5.963587 |
| GSM1297212 | Low | 1 | 1246.3477 | 1953.956 | 3200.3037 | 7.505553 |
| GSM1297214 | Low | 2 | 806.63495 | 853.87342 | 1660.5084 | 6.9067154 |
| GSM1297216 | Low | 2 | 663.65812 | 824.58296 | 1488.2411 | 8.93624 |
| GSM1297218 | Low | 2 | 334.4409 | 879.16151 | 1213.6024 | 6.444034 |
| GSM1297220 | High | 2 | 674.27327 | 720.18688 | 1394.4602 | 7.365938 |
| GSM1297222 | High | 1 | -495.6814 | -260.8813 | -756.5627 | 4.6220756 |
| GSM1297224 | High | 1 | 467.82289 | 527.54484 | 995.36773 | 7.816519 |
| GSM1297226 | Low | 2 | -977.0726 | -153.4875 | -1130.56 | 6.841899 |
| GSM1297228 | High | 2 | 395.41404 | 357.55094 | 752.96498 | 6.814438 |
| GSM1297230 | Low | 2 | 272.82837 | 404.23221 | 677.06058 | 6.351457 |
| GSM1297232 | Low | 1 | -472.2601 | 195.73347 | -276.5266 | 8.620648 |
| GSM1297234 | High | 2 | 553.1002 | 946.52283 | 1499.623 | 6.8693585 |
| GSM1297236 | Low | 1 | 952.55341 | 1817.9143 | 2770.4677 | 7.9291077 |
| GSM1297238 | Low | 1 | -438.0856 | 71.434664 | -366.6509 | 9.134397 |
| GSM1297240 | Low | 2 | -384.7488 | 551.18027 | 166.43152 | 7.322482 |
| GSM1297242 | Low | 1 | -856.8757 | 1063.971 | 207.0953 | 7.826301 |
| GSM1297244 | Low | 2 | -649.0656 | -51.66814 | -700.7337 | 8.017982 |
| GSM1297246 | Low | 1 | -636.1838 | 892.12843 | 255.94459 | 7.4116306 |
| GSM1297248 | Low | 2 | -1255.771 | -185.1943 | -1440.965 | 6.6133194 |
| GSM1297250 | High | 1 | 932.10196 | 1431.2664 | 2363.3684 | 7.2851987 |
| GSM1297252 | High | 1 | 408.25226 | 958.40327 | 1366.6555 | 7.2296767 |
| GSM1297254 | Low | 2 | -927.5155 | 120.70594 | -806.8096 | 7.1004972 |
| GSM1297256 | High | 2 | -691.4756 | -256.7722 | -948.2478 | 6.3764744 |
| GSM1297258 | Low | 1 | 647.90502 | 1685.428 | 2333.3331 | 8.214857 |
| GSM1297260 | High | 1 | -147.4574 | 557.66189 | 410.20445 | 6.7758927 |
| GSM1297262 | Low | 1 | -1029.651 | -364.5749 | -1394.226 | 7.294755 |
| GSM1297264 | High | 2 | 243.11975 | 981.8566 | 1224.9764 | 6.987484 |
| GSM1297266 | High | 1 | -247.1215 | -686.0842 | -933.2057 | 5.239849 |
| GSM1297268 | High | 2 | -1188.133 | -521.1453 | -1709.278 | 7.1749377 |
| GSM1297270 | High | 1 | 405.7525 | 943.71417 | 1349.4667 | 9.094947 |
| GSM1297272 | Low | 1 | 260.01558 | 724.83122 | 984.8468 | 9.775518 |
| GSM1297274 | Low | 1 | -215.7314 | 390.84293 | 175.11152 | 7.2680297 |
| GSM1297276 | High | 1 | 368.86554 | 1075.4391 | 1444.3046 | 7.1074176 |
| GSM1297278 | High | 1 | -472.4121 | -338.3006 | -810.7128 | 8.295756 |
| GSM1297280 | High | 1 | -255.2155 | 385.53331 | 130.3178 | 6.8543034 |
| GSM1297282 | High | 1 | 148.40831 | 1371.2953 | 1519.7036 | 8.737163 |
| GSM1297284 | High | 1 | -193.4001 | -415.7411 | -609.1413 | 6.9955316 |
| GSM1297286 | High | 1 | -642.0224 | -273.3882 | -915.4106 | 6.4062414 |
| GSM1297288 | Low | 2 | -311.5053 | -43.3944 | -354.8997 | 6.408698 |
| GSM1297290 | Low | 1 | -568.8025 | -144.6225 | -713.425 | 8.877113 |
| GSM1297292 | Low | 2 | 231.66763 | 982.01191 | 1213.6795 | 9.007935 |
| GSM1297294 | Low | 1 | -316.0266 | -106.7507 | -422.7774 | 7.012195 |
| GSM1297296 | High | 1 | 242.38755 | 1168.9293 | 1411.3168 | 7.000974 |
| GSM1297298 | High | 2 | 1107.4477 | 1779.2428 | 2886.6906 | 8.741956 |
| GSM1297300 | Low | 2 | 188.58429 | 771.59473 | 960.17903 | 9.281837 |
| GSM1297302 | High | 2 | 12.130557 | 1245.726 | 1257.8566 | 10.378348 |
| GSM1297304 | High | 1 | 145.24163 | 628.53679 | 773.77841 | 6.287255 |
| GSM1297306 | Low | 2 | 243.01142 | 498.33474 | 741.34617 | 8.085388 |
| GSM1297308 | High | 1 | 190.21134 | 1017.3135 | 1207.5248 | 6.7718797 |
| GSM1297310 | Low | 2 | -466.848 | -232.7944 | -699.6424 | 5.996352 |
| GSM1297312 | Low | 2 | -417.4772 | 453.32107 | 35.843861 | 7.1526284 |

ESCC, esophageal squamous cell carcinoma.
